# SARS-CoV-2 Plasma Cells are Not Durably Established in the Bone Marrow Long-Lived Compartment after mRNA Vaccination

**DOI:** 10.1038/s41591-024-03278-y

**Published:** 2024-09-27

**Authors:** Doan C. Nguyen, Ian T. Hentenaar, Andrea Morrison-Porter, David Solano, Natalie S. Haddad, Carlos Castrillon, Martin C. Runnstrom, Pedro A. Lamothe, Joel Andrews, Danielle Roberts, Sagar Lonial, Ignacio Sanz, F. Eun-Hyung Lee

**Affiliations:** 1 Division of Pulmonary, Allergy, Critical Care, and Sleep Medicine, Department of Medicine, Emory University, Atlanta, GA, United States; 2 Division of Rheumatology, Department of Medicine, Emory University, Atlanta, GA, United States; 3 Department of Hematology and Medical Oncology, Winship Cancer Institute, Emory University, Atlanta, GA, United States; 4 Lowance Center for Human Immunology, Emory University, Atlanta, GA, United States

## Abstract

SARS-CoV-2 mRNA vaccines are effective at protecting from severe disease, but the protective antibodies wane rapidly even though SARS-CoV-2-specific plasma cells can be found in the bone marrow (BM). To explore this paradox, we enrolled 19 healthy adults at 2.5–33 months after receipt of a SARS-CoV-2 mRNA vaccine and measured influenza-, tetanus-, or SARS-CoV-2-specific antibody-secreting cells (ASC) in long-lived plasma cell (LLPC) and Non-LLPC subsets within the BM. Only influenza- and tetanus-specific ASC were readily detected in the LLPC whereas SARS-CoV-2 specificities were mostly absent. The ratios of Non-LLPC:LLPC for influenza, tetanus, and SARS-CoV-2 were 0.61, 0.44, and 29.07, respectively. In five patients with known PCR-proven history of recent infection and vaccination, SARS-CoV-2-specific ASC were mostly absent from the LLPC. We show similar results with measurement for secreted antibodies from BM ASC culture supernatant. While serum IgG titers specific for influenza and tetanus correlated with IgG LLPC, serum IgG levels for SARS-CoV-2, which waned within 3–6 months after vaccination, were associated with IgG Non-LLPC. In all, our studies suggest that rapid waning of SARS-CoV-2-specific serum antibodies could be accounted for by the absence of BM LLPC after these mRNA vaccines.

## Introduction

As of August 2024, SARS-CoV-2 has infected over 776 million people worldwide and killed 7.1 million, including 1.2 million in the United States alone^1^. While the original wildtype SARS-CoV-2 primary vaccine series and boosters have been effective against severe disease, hospitalization, and death, protection by sterilizing immunity against infection or transmission has not been demonstrated. SARS-CoV-2 vaccines appear to provide lasting T cell responses; however, waning neutralizing antibody levels within 3–6 months results in breakthrough infection (BTI) or reinfections with the same strain^2–4^. Therefore, we asked whether subjects after SARS-CoV-2 vaccination develop spike-specificity in the long-lived plasma cell (LLPC) subset (CD19^−^CD38^hi^CD138^+^) of the human bone marrow (BM)^5^. For clarity, the term ASC refers to all antibody-secreting cells (ASC), which include early-minted ASC (oftentimes referred to as plasmablasts^6^) and more mature ASC known as plasma cells that can contain LLPC.

Early in the COVID-19 pandemic, studies reported that SARS-CoV-2 spike IgG ASC were readily identified in the BM after SARS-CoV-2 infection^7^ or mRNA vaccination^8^, and in non-human primates after SARS-CoV-2 spike protein vaccination^9^, suggesting long-lived humoral protection without evidence of longitudinal serologic data. Interestingly, BM ASC compartments can be quite diverse, comprising of early-minted ASC (new arrivals) of which many may die, while some progressively mature into LLPC^10–15^. How LLPC are generated is not entirely clear, but after vaccination, the majority of ASC released from secondary lymph nodes are destined to undergo apoptosis unless they finally arrive in the specialized BM survival niches filled with mesenchymal stromal cells and myeloid cells. This niche provides important factors for ASC survival and maturation such as IL-6 and APRIL^16,17^. These new arrivals can further differentiate into a mature long-lived phenotype (CD19^−^CD138^+^), which secretes neutralizing antibodies for decades^10,11^. Although the human BM is a reservoir of LLPC, new arrivals, including CD19^+^CD138^−^ and intermediate phenotypes of CD19^+^CD138^+^ ASC, make it quite heterogenous^12,18^, such that mere presence to this locale may not reflect durability.

Tetanus vaccination generates antigen-specific BM LLPC and affords safeguards for decades with a serologic half-life of 10 years^10,19^. For influenza, humoral immune protection provided by influenza vaccines typically wanes within 4–6 months^20^. Infants may have preexisting maternally derived anti-influenza antibodies although they wane over the first six months of life^21^. Unvaccinated individuals are estimated to have their first influenza infection within five years of birth^22^ and to be infected with a new influenza virus strain every 3–7 years^23^. Furthermore, newly induced immune responses are enhanced owing to cross-reactive antibodies from infections and reinfections with antigenically similar influenza virus strains^24–28^. Importantly, natural influenza infection generates long-lasting humoral immunity to the infecting strain, as shown in elderly adults who maintained neutralizing antibodies to the 1918 Spanish influenza virus nearly 90 years after the primary infection^29^.

Here, we measure SARS-CoV-2 spike-specific ASC in multiple BM compartments up to 33 months after SARS-CoV-2 mRNA vaccination and compare them to well-known long-lived responses such as tetanus- and influenza-specific ASC and show the absence of SARS-CoV-2-specific ASC in the BM LLPC. This finding provides a mechanistic explanation for the short duration of systemic antibody responses to SARS-CoV-2 mRNA vaccines.

## Results

### Demographic and clinical characteristics

**Fig. 1.**
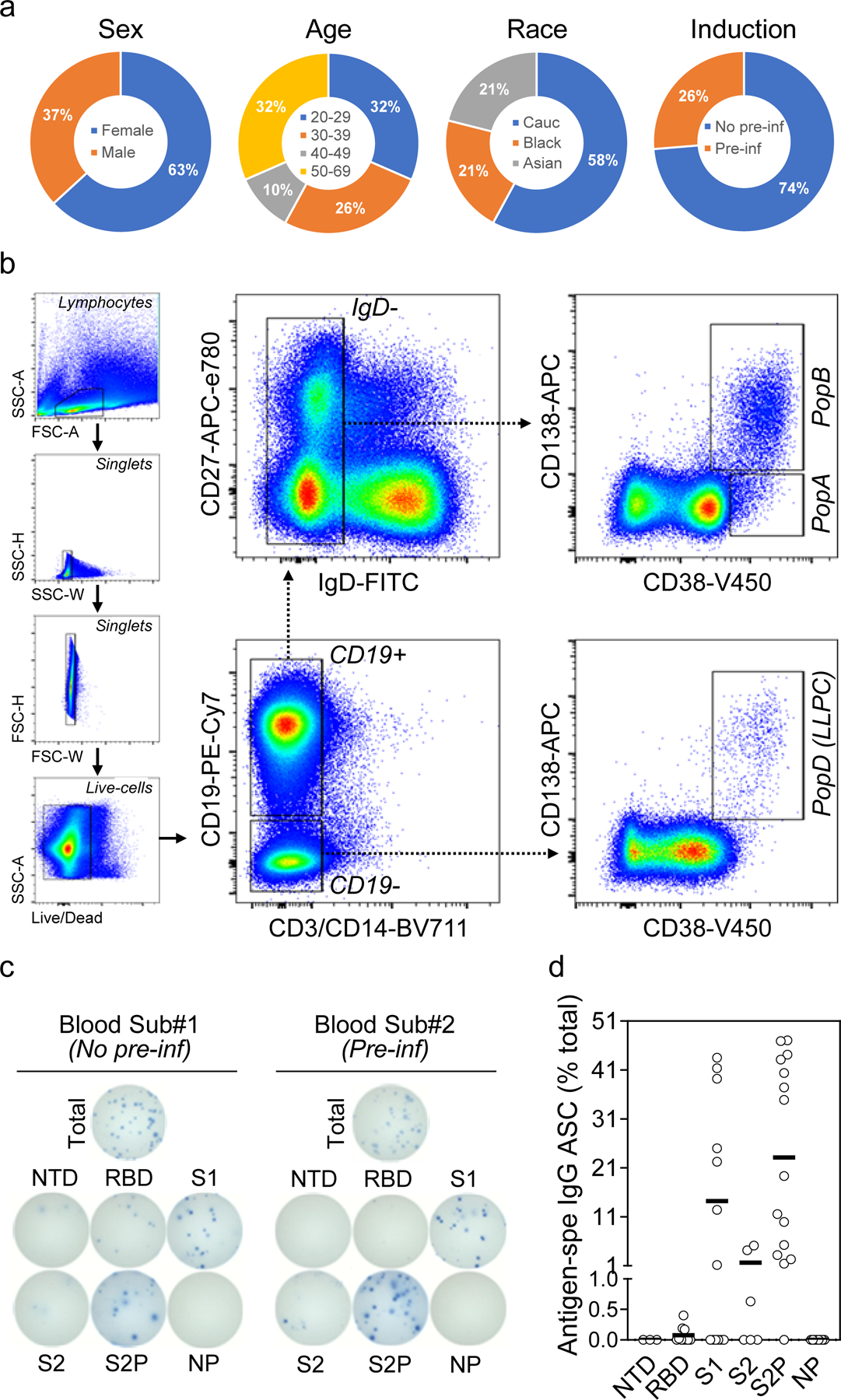
Demographics of the 19 BM subject cohort (**a**). Age, years; Cauc, Caucasian; Pre-inf, previous (COVID-19) infection. **General FACS gating strategy used for sorting BM ASC subsets** (**b**). BMMC were first gated for lymphocytes, singlets, and viable cells (based on their FSC/SSC and Live/Death properties). CD3 and CD14 were then used as dump markers to capture CD19^+^ and CD19^−^ B cell populations. Subsequent sub-gating from CD19^+^ population on the IgD^−^ fraction (versus CD27) and using CD138 versus CD38 allowed for breaking down BM ASC populations into three subsets of interest: PopA (CD19^+^CD38^hi^CD138^−^), PopB (CD19^+^CD38^hi^CD138^+^), and PopD (LLPC; CD19^−^CD38^hi^CD138^+^). **S2P was most sensitive to capture SARS-CoV-2-specific blood ASC isolated after SARS-CoV-2 mRNA vaccines** (**c**,**d**). Representative ELISpot scanned images (left, a vaccinated subject without previous COVID-19 infection; right, a vaccinated subject with previous COVID-19 infection) (**c**). The numbers of input ASC that were incubated for total IgG: ~687 (left) or ~522 (right), and for antigen-specific ASC IgG: ~2,062 (left) or ~1,566 (right). Each circle represents an individual vaccine subject. ~: counts provided by the sorters. Blood ASC from subjects collected at the peak time of response (which is 5–7 days post-vaccine). Frequencies (%) of antigen-specific IgG ASC per total IgG ASC (**d**). Data were generated from 3, 10, 13, 6, 15, and 8 different SARS-CoV-2 vaccinated subjects for SARS-CoV-2 antigens NTD, RBD, S1, S2, S2P, and NP, respectively. Statistics were assessed using Student’s t-test (two-tailed unpaired t-test) in Excel (Microsoft) and differences were considered significant at p values less than 0.05. For additional antigen selection and validation, see Extended Data Figure 2.

From May 2021 until March 2024, we enrolled 19 healthy adults aged of 20–65 years old (Fig. 1a). The subjects were recruited for BM aspirates 2.5–33 months after receiving the first dose of SARS-CoV-2 mRNA vaccines. All received a total of two to five vaccine doses, and BM aspirates were obtained 0.5–21 months after receiving the last booster (the third, fourth, or fifth dose) (Table 1). One subject provided three longitudinal BM samples over a period of 21 months and a second subject had two aspirates over six months, resulting in a total of 22 BM aspirates. Five subjects reported infection with SARS-CoV-2 1–16 months prior to the BM collection, of which three subjects had infection once and two had two PCR-proven SARS-CoV-2 infections. These infections occurred 1–15.5 months after receiving the most recent vaccine dose. All 19 individuals received the quadrivalent influenza vaccine within 1–12 months (relative to the time of each BM aspirate) and one was delayed for one year due to the COVID-19 pandemic. All received the childhood series of the tetanus toxoid vaccine with recent boosters ranging from one month to 24 years from the time of BM aspirates.

### BM ASC subsets and antigen optimization

**Extended Data Fig. 1:**
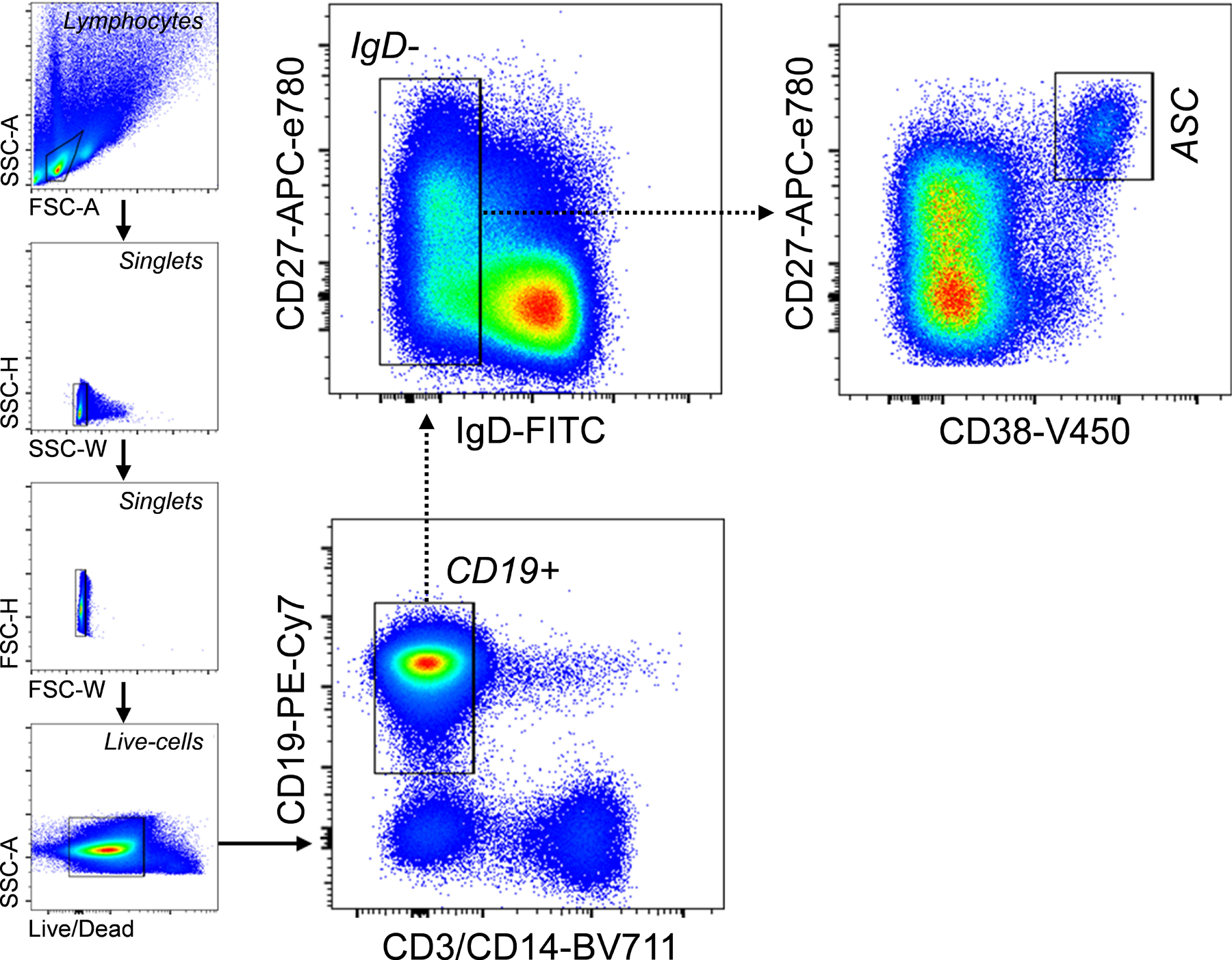
General FACS gating strategy used for sorting blood ASC.</P/>PBMC were first gated for lymphocytes, singlets, and viable cells (based on their FSC/SSC and Live/Death properties). CD3 and CD14 were then used as dump markers to capture CD19+ and CD19- B cell populations. Subsequent sub-gating using CD38 versus CD27 on the IgD- fraction (of CD19+ population) allows for sorting for blood ASC (CD27hiCD38hi). See Methods for antibody panels.

**Extended Data Fig. 2:**
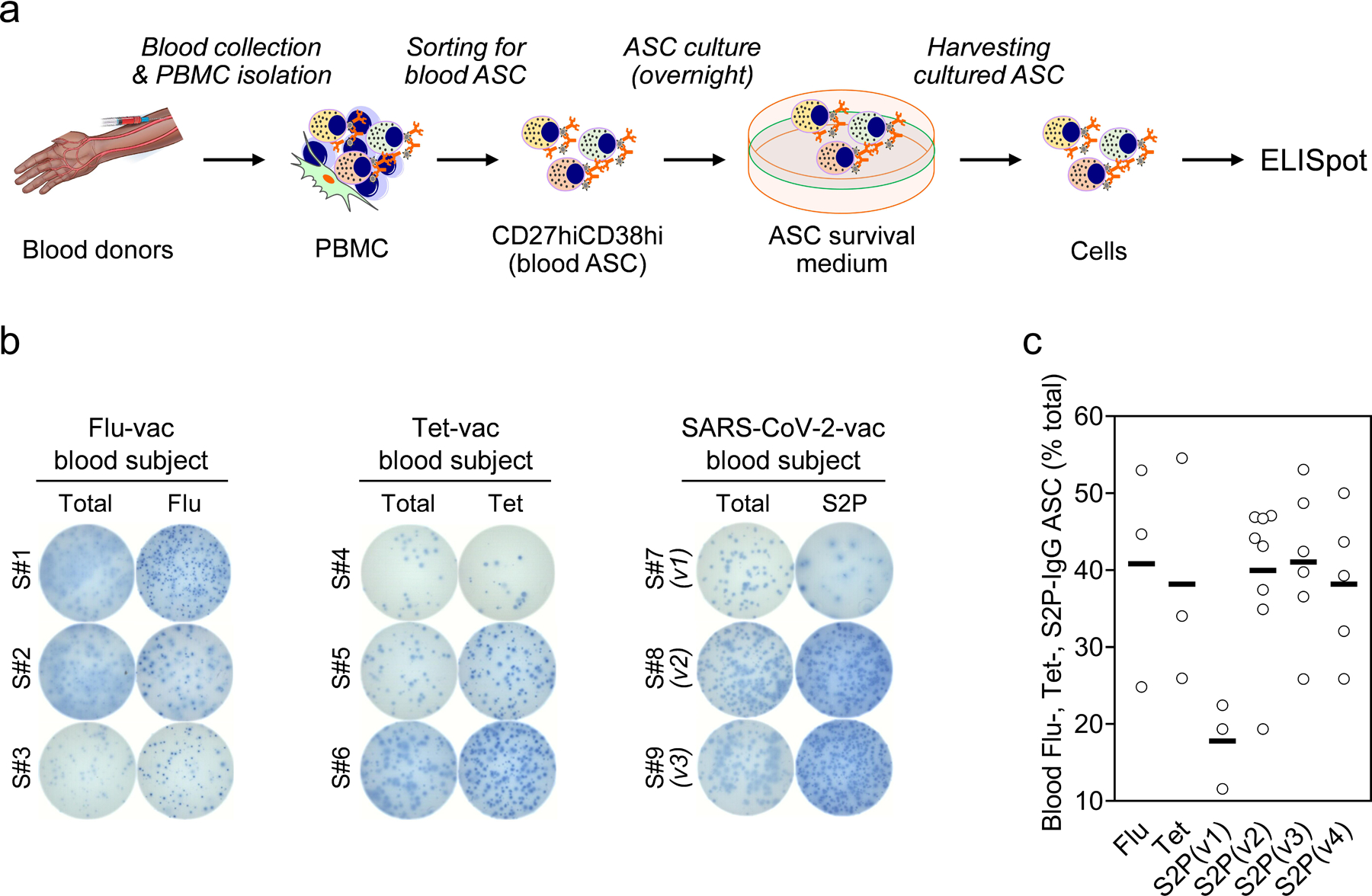
Assessment of vaccine-specific ASC and validation of vaccine specificities with blood ASC.</P/>(a) Summary of the techniques and the experimental designs. From the cultures of blood ASC, the cells were collected and ELISpot-quantitated for validating vaccine specificities. (b) Representative ELISpot scanned images shown. Blood ASC from subjects at the peak (5–7 days post-vaccine) assayed for Flu-, Tet-, and S2P-specific IgG secretion. The numbers of input ASC that were incubated were ~894, ~1,124, and ~796 (total), and ~4,471, ~4,496, and ~2,388 (Flu-specific) for S#1, S#2, and S#3, respectively (far left); ~1K, ~1K, and ~1K (total), and ~3K, ~4K, and ~4K (Tet-specific) for S#4, S#5, and S#6, respectively (left); and ~712, ~1,415, and ~1,386 (total), and ~2,139, ~4,245, and ~5,544 (S2P-specific) for S#7, S#8, and S#9, respectively (right). (c) Each circle represents an individual vaccinee. Data were generated from 3, 3, 3, 8, 6, and 5 different vaccinated subjects for Flu, Tet, S2P (v1), S2P (v2), S2P (v3), and S2P (v4), respectively. S: subject; ~: counts provided by the sorters; K: 1,000; vac: vaccinated; Flu: influenza; Tet: tetanus; v: (SARS-CoV-2 mRNA) vaccine dose. All ASC assayed at day 1 in culture.

BM ASC subsets were FACS-sorted according to surface expression of CD19, CD38, and CD138^10^ (Fig. 1b). To overcome the problem with the rapid death of ASC *ex vivo*^16^, we rested the ASC overnight in a new human *in vitro* plasma cell survival system which is capable of maintaining human ASC viability for months^16^. Since we had previously localized the BM LLPC compartment into PopD (CD19^−^CD38^hi^CD138^+^)^10^, this population was sorted out of total BM ASC together with Non-LLPC subsets: PopA (CD19^+^CD38^hi^CD138^−^) and PopB (CD19^+^CD38^hi^CD138^+^). All were tested for total IgG secretion as well as, influenza- (Flu-), tetanus toxoid- (Tet-), and SARS-CoV-2-specific IgG secretion by bulk ELISpots. To optimize antigen detection for the BM ASC ELISpot assays, we collected early-minted blood ASC (CD27^hi^CD38^hi^; Extended Data Figure 1) 6–7 days after Flu, Tet, or SARS-CoV-2 vaccination, which is the peak time for enrichment of vaccine-specific ASC in the blood after secondary immunization^30,31^, and performed ELISpots (Extended Data Figure 2a). Of the SARS-CoV-2 antigens (S1, S2, RBD, S2P, NTD, and NP proteins), S2P, a prefusion-stabilized spike trimer^32^, generated the highest frequency, followed by S1 (with no significant difference; *p*=0.21) (Fig. 1c,d), and so S2P was selected for the BM ASC ELISpot assays. We also validated the quadrivalent Flu vaccine (seasons of 2019–20 to 2023–24) as the Flu antigen and Tet antigen (Extended Data Figure 2b,c), using blood ASC at day 6–7, the peak of the respective ASC vaccine responses^30,31^.

### Absence of SARS-CoV-2-specific IgG BM LLPC

**Fig. 2.**
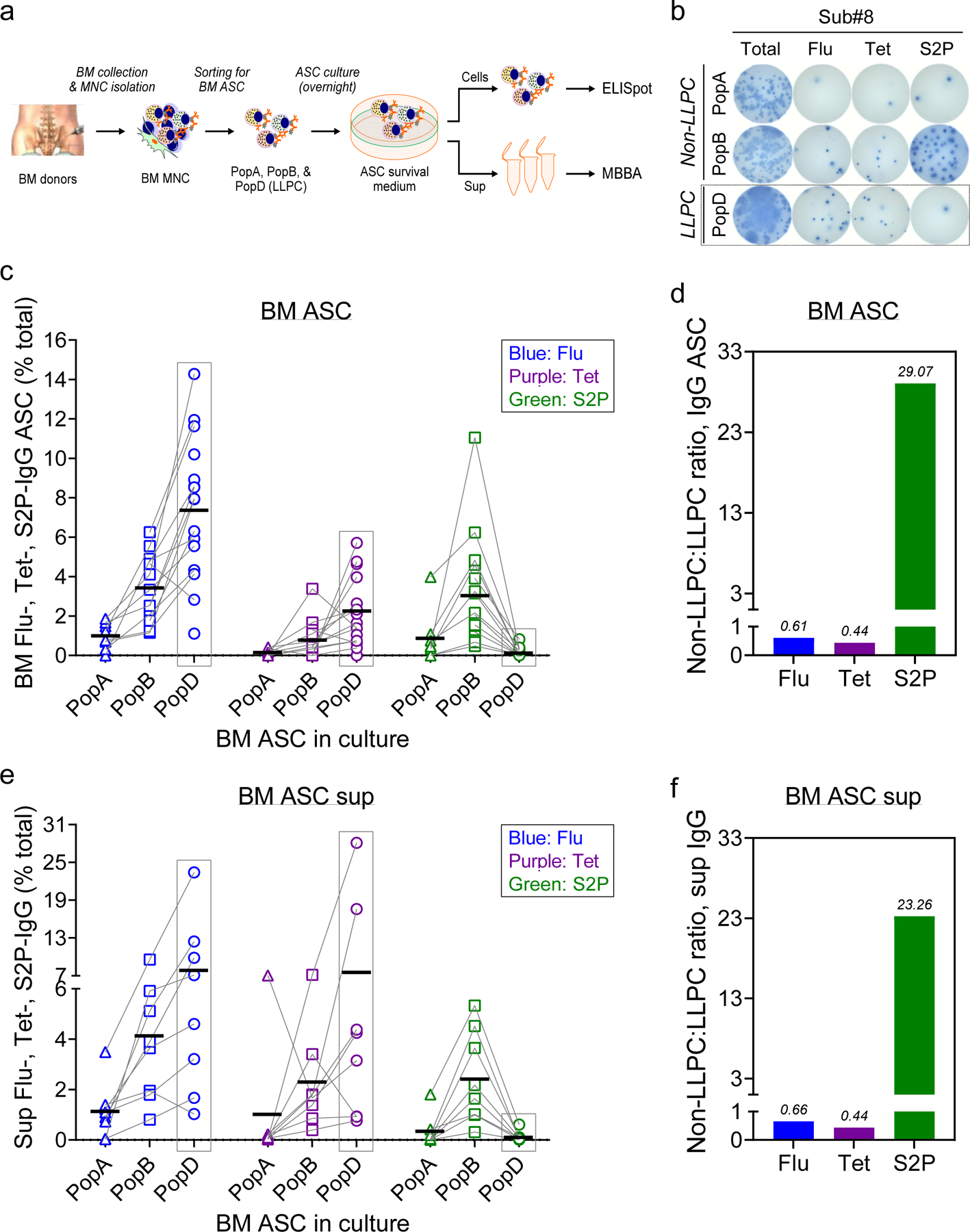
Absence of SARS-CoV-2 BM IgG LLPC after SARS-CoV-2 mRNA vaccines by detection of ASC and secreted IgG in the BM ASC culture supernatants. Summary of the techniques and the experimental designs for detection of total, Flu, Tet, and S2P ASC and secreted IgG by ELISpots and MBBA, respectively (**a**). Representative ELISpot scanned images (**b**). The numbers of input ASC that were incubated were ~52K, ~12.1K, and ~10.1K for PopA, PopB, and PopD, respectively. Each symbol represents an individual vaccine subject for total IgG and antigen-specific ASC from PopA, PopB, and PopD. ELISpots measuring BM IgG ASC specific for Flu, Tet, and S2P (**c**). Data were generated from 8, 15, and 17 different SARS-CoV-2 vaccinated subjects for PopA, PopB, and PopD, respectively. For individual ratios and statistic comparisons between any two antigens for any subset or between any two subsets for any antigen, see Supplementary Table 1 and Supplementary Table 2, respectively. Fold difference (ratios) when comparing different vaccine specificities between Non-LLPC (combined PopA and PopB) versus LLPC (PopD) (**d**). MBBA measuring IgG specific for Flu, Tet, and S2P (normalized to total IgG) from culture supernatant of PopA, PopB, and PopD (**e**). Supernatant preps were collected from 18–24-hour cultures of BM ASC after revival from the FACS sorters and were quantified for total IgG and vaccine-specific IgG in neat (undiluted). Data were generated from eight different SARS-CoV-2 vaccinated subjects. For individual ratios and statistic comparisons between any two antigens for any subset or between any two subsets for any antigen, see Supplementary Table 1 and Supplementary Table 2, respectively. Fold difference (ratios) when comparing normalized vaccine-specific IgG in the supernatants from the culture of Non-LLPC (combined PopA and PopB) versus LLPC (PopD) (**f**). For ratio calculation, see Methods. For IgG standard versus MFI curve, see Extended Data Figure 4. Sub: subject; ~: counts provided by the sorters; K: 1,000; LLPC: long-lived plasma cell (dotted boxes in **b**, **c**, and **e**); Flu: influenza; Tet: tetanus; Sups: BM ASC culture supernatant preps; MBBA: multiplex bead binding assay. For details of subjects and samples, see Table 1.

Since BM aspirates can yield variable cell numbers, we included BM aspirates with >3,000 sorted cells in each of the three ASC populations, cultured the cells overnight in a specialized *in vitro* BM mimetic system^16^, and performed bulk ELISpots (Fig. 2a; see also Methods). Among the BM subjects, sufficient cells to confidently measure vaccine-specificities within PopA, PopB, and PopD were obtained from 8, 15, and 17 individuals. As previously shown, all BM ASC subsets had detectable total IgG ASC. Similar to previous reports^10^, PopD contained the highest percentage of Flu and Tet IgG ASC per total IgG ASC: mean 7.3% (7.31±3.51) and 2.1% (2.14±1.70) respectively (Fig. 2b,c). PopB was readily populated with Flu and Tet IgG ASC: mean 3.4% (3.43±1.68) and 0.8% (0.77±0.87) respectively, while PopA had the lowest frequencies: mean 1% (1.0±0.66) and 0.2% (0.17±0.17) respectively. Strikingly, within the same subjects, we could rarely detect S2P ASC in PopD: mean 0.1% (0.14±0.23). In contrast, the S2P specificity was readily found in PopB and PopA at frequencies comparable to Tet and Flu: mean 3.1% (3.13±2.82) and 0.9% (0.89±1.3) respectively.

Although the frequencies of Tet IgG ASC in PopA versus PopB showed no statistically significant difference, the frequencies of Flu IgG were higher in PopB over PopA. For both Flu and Tet IgG ASC, the frequencies in PopD were always higher than in PopB. In contrast, the S2P IgG ASC frequencies were always significantly lower in PopD compared to PopB (Fig. 2b,c). On average, the fold changes of IgG ASC specificities within PopD were 52.8 for Flu:S2P and 15.5 for Tet:S2P (Supplementary Table 1). In comparison, the fold changes of IgG ASC specificities within PopB were 1.1 for Flu:S2P and 0.3 for Tet:S2P. For S2P specificity, the fold changes of PopA:PopD was 6.4 and of PopB:PopD was 22.6 (Supplementary Table 2). In comparison, for Flu or Tet specificities, these fold changes were ≤0.47. Overall, the ratios of Non-LLPC:LLPC for Flu, Tet, and S2P were 0.61, 0.44, and 29.07, respectively (Fig. 2d). Thus, S2P IgG ASC are largely excluded from PopD.

### Absence of SARS-CoV-2-specific IgA BM LLPC

**Extended Data Fig. 3:**
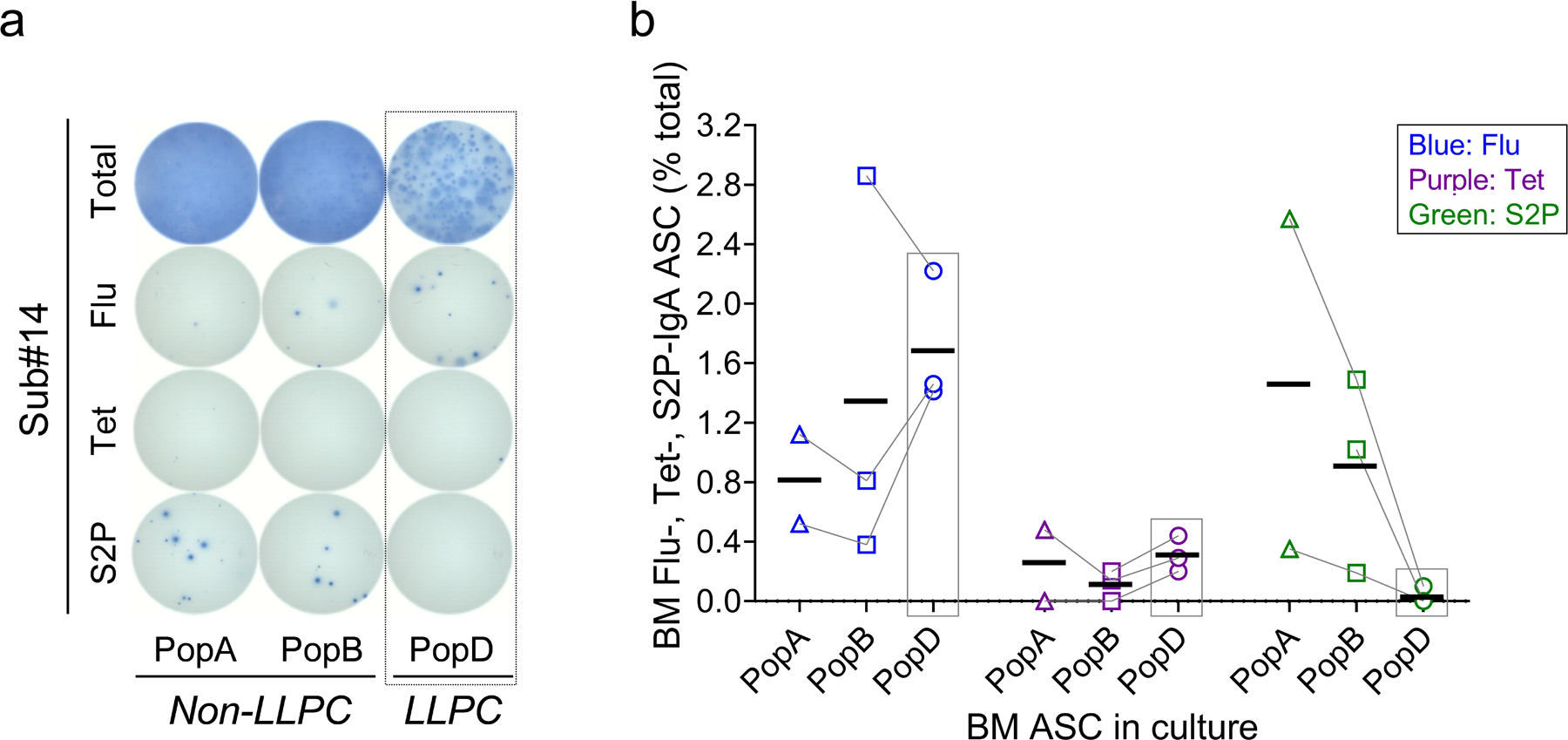
Exclusion of S2P BM IgA LLPC in SARS-CoV-2 mRNA vaccinees.</P/>(a) Representative ELISpot scanned images. The numbers of input ASC that were incubated were ~9.6K, ~3.7K, and ~1.2K (total) and ~58K, ~22K, and ~7.2K (vaccine-specific) for PopA, PopB, and PopD, respectively. (b) Each symbol represents an individual vaccinee. Data were generated from 2, 3, and 3 different SARS-CoV-2 vaccinated subjects for PopA, PopB, and PopD, respectively. Statistics were assessed using Student’s t-test (two-tailed unpaired t-test) in Excel (Microsoft) and differences were considered significant at p values less than 0.05. ~: counts provided by the sorters; K: 1,000; LLPC: long-lived plasma cell (dotted boxes); Flu: influenza; Tet: tetanus. All ASC assayed at day 1 in culture. For individual ratios and statistic comparisons between any two antigens for any subset or between any two subsets for any antigen, see Supplementary Table 3 and Supplementary Table 4, respectively.

Similar to IgG ASC, the frequencies of Flu and Tet IgA ASC were highest in PopD with a mean of 1.7% (1.70±0.45) and 0.3% (0.31±0.12) respectively, while frequencies in PopA and PopB were lower: for Flu, mean 0.8% (0.82±0.43) and 1.4% (1.35±1.32) respectively, and for Tet, 0.2% (0.24±0.34) and 0.1% (0.11±0.10) respectively (Extended Data Figure 3a,b). Consistent with previous studies^33^, these results may be explained by the predominance of IgG responses to the intramuscular tetanus vaccine. S2P IgA ASC were also detected predominantly in PopA and PopB: a mean of 1.5% (1.46%±1.57) and 0.9% (0.90±0.66) respectively, and were virtually absent in PopD: a mean of 0.03% (0.03%±0.06) (Extended Data Figure 3b). On average, the fold changes of IgA ASC specificities within PopD were 50.9 for Flu:S2P and 9.3 for Tet:S2P (Supplementary Table 3). For S2P specificity, the fold changes of PopA:PopD was 43.8 and of PopB:PopD was 27.0 (Supplementary Table 4). Thus, similar to IgG ASC, other class-switched isotypes such as S2P IgA ASC are also mostly excluded from PopD (albeit small sample size).

### Absence of SARS-CoV-2-specific IgG in LLPC culture supernatant

**Extended Data Fig. 4:**
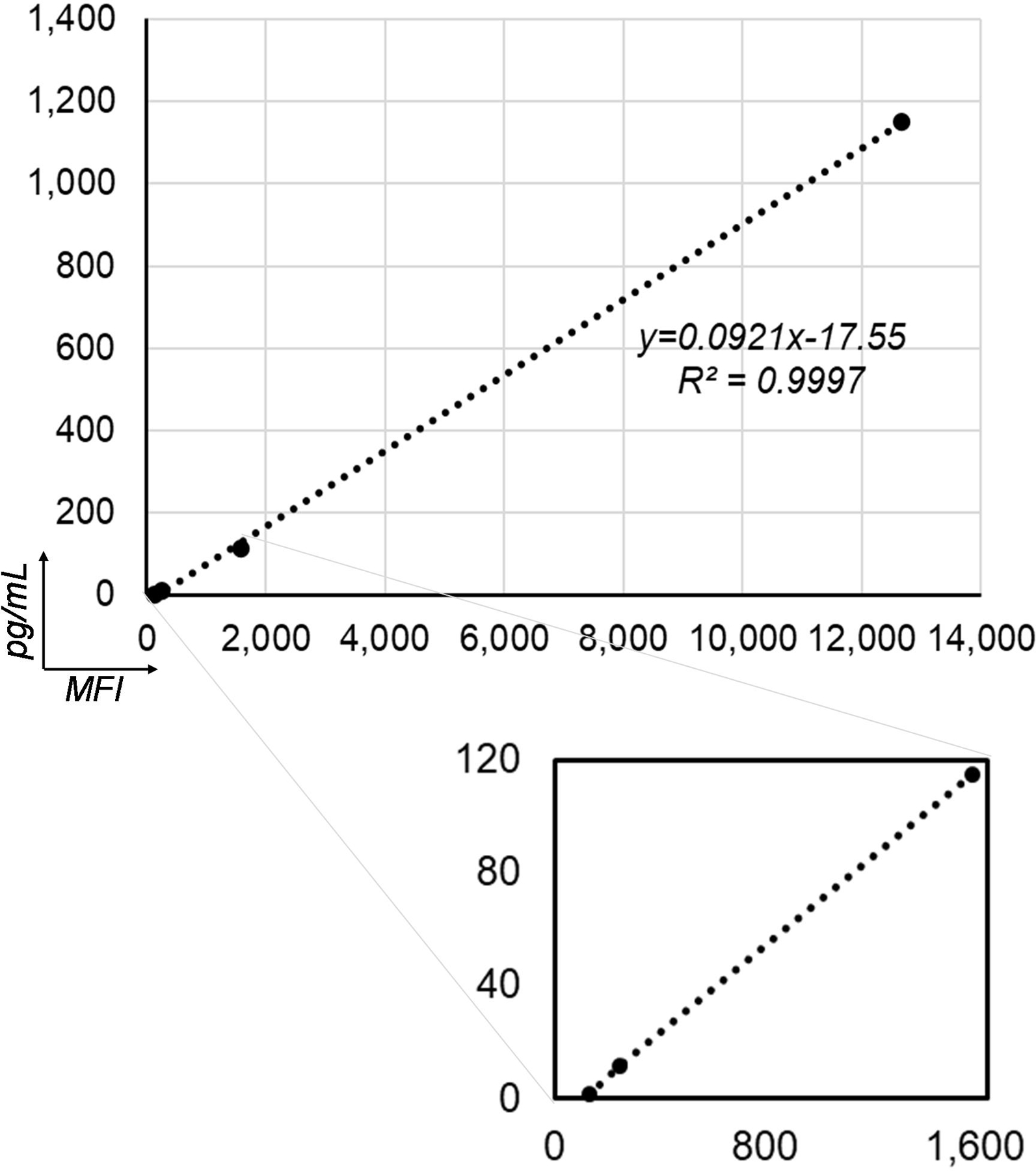
The human IgG standardized concentrations versus MFI values.</P/>The displayed equation was used to normalize MFI values for detection of antibodies in the culture supernatants of each BM ASC subset.

To validate the antigen-specific ELISpot results, we measured secreted IgG from BM ASC subsets (Fig. 2a; see also Methods). Briefly, from eight individuals that yielded sufficient sorted cells for all BM ASC subsets (PopA, PopB, and PopD), we cultured ASC in a specialized *in vitro* BM mimetic system overnight^16^ and measured the cultured supernatants for secreted IgG specific for Flu, Tet, and S2P by multiplex bead-binding assays^34^ (Extended Data Figure 4). The results were similar to the ELISpot: the percentages of Flu and Tet IgG per total IgG were highest in PopD (mean 7.92±7.41 and 7.51±9.98, respectively) compared to PopB (mean 4.09±2.81 and 2.30±2.14, respectively) or PopA (mean 1.12±1.08 and 0.97±2.46, respectively) (Fig. 2e). In contrast, the percentage of S2P IgG per total IgG was lower in PopD (mean 0.12±0.20) compared to PopA (mean 0.31±0.62) and especially to PopB (mean 2.46±1.83).

Of eight individuals, the fold change in PopD for Flu:S2P was 66.5 and for Tet:S2P was 63.1 (Supplementary Table 1). In comparison, the fold change within PopB for Flu:S2P was 1.7 and for Tet:S2P was 0.9, demonstrating similar quantities of IgG to Flu, Tet, and S2P in PopB. Within the S2P specificity, the fold changes of S2P IgG levels in the BM culture supernatants for PopA:PopD and PopB:PopD were 2.6 and 20.1, respectively (Supplementary Table 2). In comparison, for Flu or Tet specificities, these fold changes were ≤0.31. Ultimately, using this method of measuring secreted antibodies from the cultured BM ASC, the ratios of Non-LLPC:LLPC for Flu, Tet, and S2P from BM ASC culture supernatant were 0.66, 0.44, and 23.26, which was similar to the ELISpot results (Fig. 2f). In all, we validate the antigen specificities observed by the ELISpots using our novel *in vitro* plasma cell culture methods which also showed exclusion of SARS-CoV-2-specific ASC in PopD.

### No correlation of S2P BM ASC responses and time from first vaccine

**Extended Data Fig. 5:**
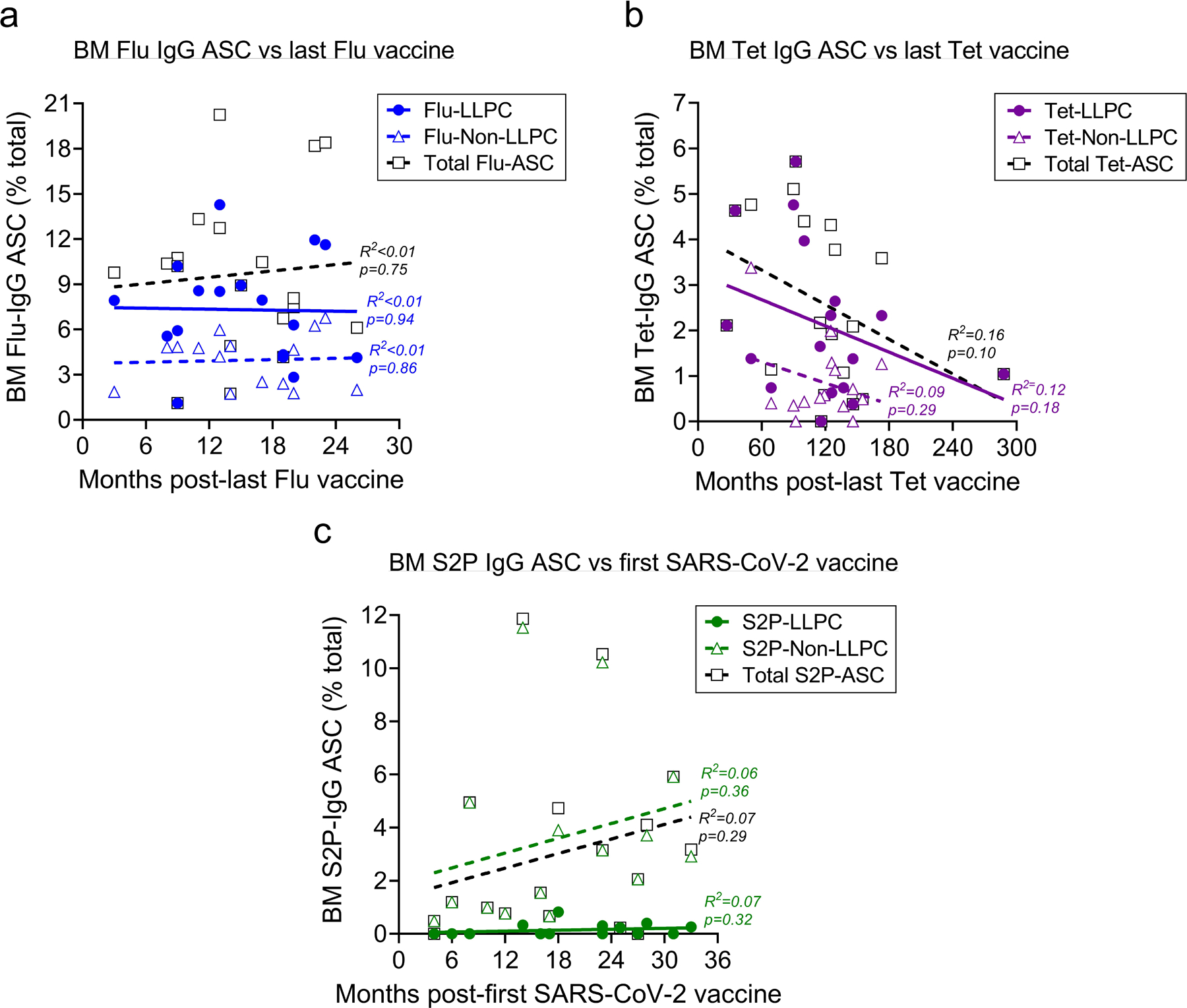
No correlation between vaccine-specific IgG BM ASC responses and the time windows of the vaccine.</P/>BM (a) Flu, (b) Tet, and (c) S2P IgG LLPC, Non-LLPC, and total ASC responses in all examined subjects following the vaccine exposure time (time since the most recent (a) Flu, (b) Tet, or (c) the first SARS-CoV-2 vaccine). Data were generated from 17, 15, and 19 different SARS-CoV-2 vaccinated subjects for BM LLPC, Non-LLPC, and total ASC, respectively. Correlations were assessed using simple linear regression analysis performed with GraphPad Prism (GraphPad Software). The exact p values for vaccine-specific LLPC, Non-LLPC, and total ASC are 0.9397, 0.8563, and 0.7455, respectively (a); 0.1806, 0.2898, and 0.0952, respectively (b); and 0.3202, 0.3635, and 0.2862, respectively (c). Subjects yielding sufficient ASC for LLPC and Non-LLPC subsets included.

**Extended Data Fig. 6:**
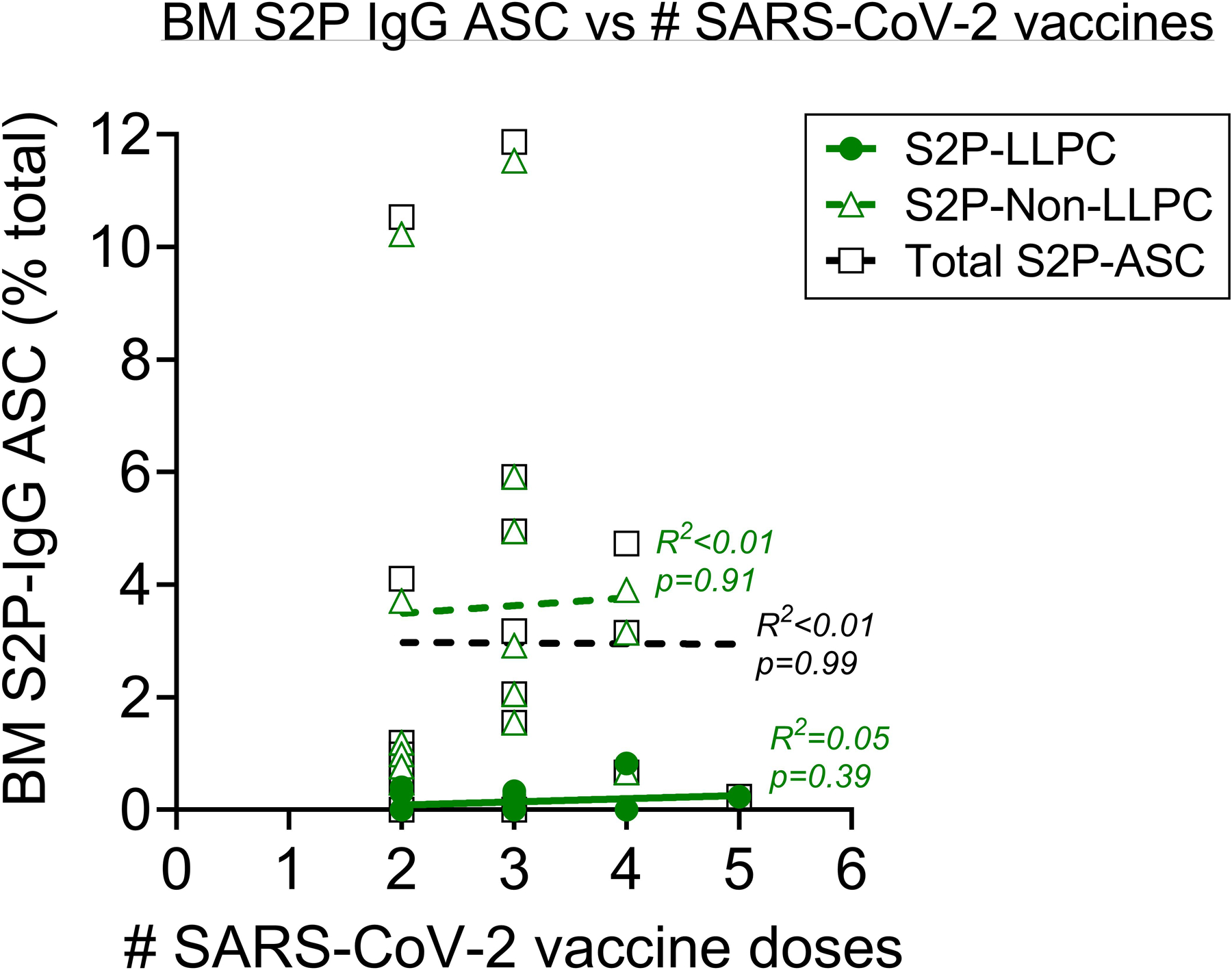
No correlation between S2P IgG BM ASC responses and the number of SARS-CoV-2 vaccine doses.</P/>BM S2P IgG ASC responses in all examined subjects stratified by the number of SARS-CoV-2 vaccine doses (prior to BM aspiration). Data were generated from 17, 15, and 19 different SARS-CoV-2 vaccinated subjects for BM LLPC, Non-LLPC, and total ASC, respectively. The exact p values for S2P LLPC, Non-LLPC, and total ASC are 0.3929, 0.9110, and 0.9912, respectively. Subjects yielding sufficient ASC for LLPC and Non-LLPC subsets included.

**Extended Data Fig. 7:**
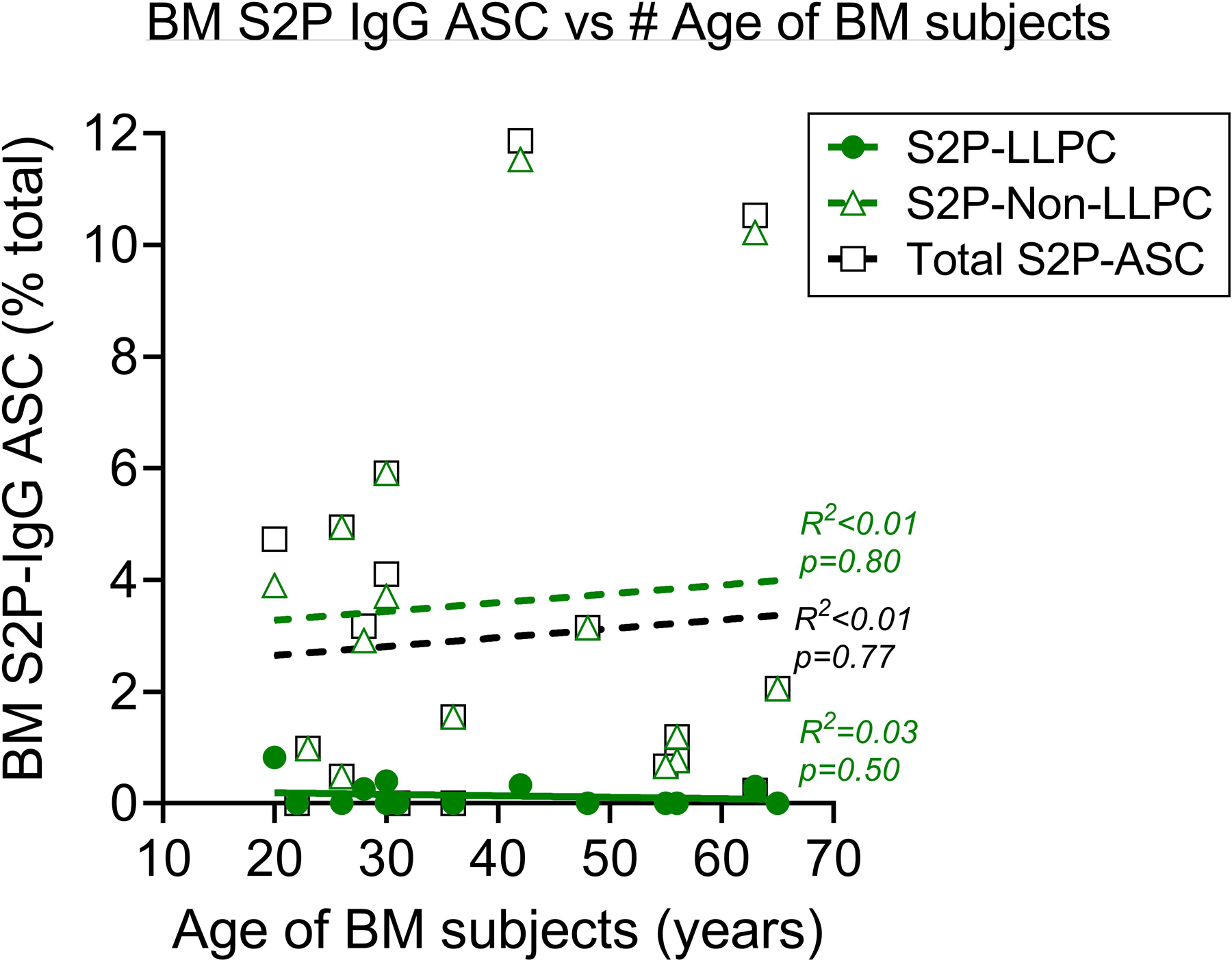
No correlation between S2P IgG BM ASC responses and age of the subjects.</P/>BM S2P IgG ASC responses in all examined subjects stratified by age of the subjects at the time of BM collection. Data were generated from 17, 15, and 19 different SARS-CoV-2 vaccinated subjects for BM LLPC, Non-LLPC, and total ASC, respectively. The exact p values for S2P LLPC, Non-LLPC, and total ASC are 0.4950, 0.7976, and 0.7699, respectively. Subjects yielding sufficient ASC for LLPC and Non-LLPC subsets included.

Because the time from vaccination to BM aspiration varied among the subjects, we compared the time from the last Flu and Tet vaccine as well as from the first SARS-CoV-2 vaccine with the vaccine-specific BM ASC responses. For Flu and Tet, we saw no correlation between the frequencies of vaccine-specific BM ASC subsets (including PopD) with the time windows since the last Flu or Tet vaccine (R^2^≤0.16, p≥0.10; Extended Data Figure 5a,b). Similarly, the S2P BM LLPC and Non-LLPC frequencies did not correlate with the time from the first vaccine (R^2^≤0.07, p≥0.29; Extended Data Figure 5c), the number of vaccine doses (R^2^≤0.05, p≥0.39; Extended Data Figure 6), or age of BM subjects (R^2^≤0.03, p≥0.50; Extended Data Figure 7). These results suggest that more time since SARS-CoV-2 mRNA vaccination or more vaccine doses does not necessarily promote more S2P PopD responses (in our small cohort).

### No differences in BM ASC after vaccine or vaccine with infection

**Extended Data Fig. 8:**
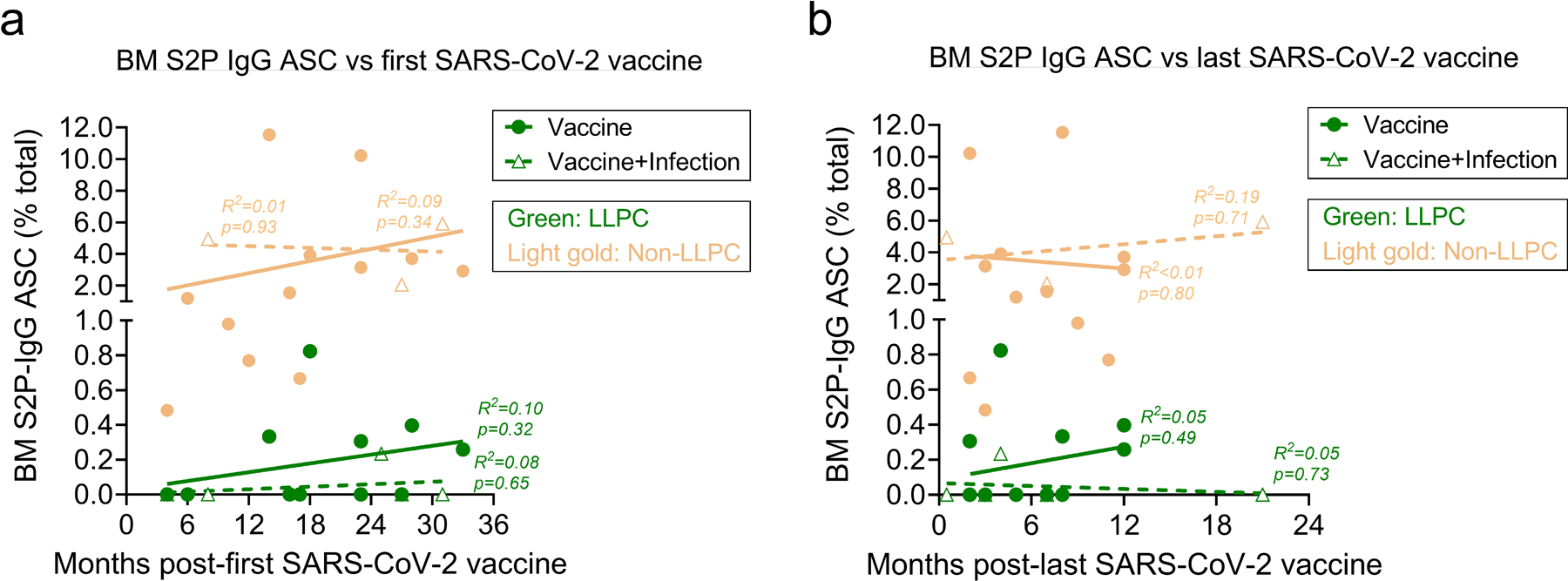
No correlation between S2P IgG BM ASC responses and the time windows of the SARS-CoV-2 vaccine in both vaccinees and infected vaccinees (Vaccine+Infection).</P/>BM S2P IgG SC responses in vaccinated versus hybrid subjects stratified by time since (a) the first or (b) the most recent (prior to BM aspiration) SARS-CoV-2 vaccine. The exposure time for infection in the hybrid subjects not shown. For BM LLPC, data were generated from 12 and 5 different subjects of Vaccine and of Vaccine+Infection, respectively. For BM Non-LLPC, data were generated from 12 and 3 different subjects of Vaccine and of Vaccine+Infection, respectively. The exact p values for Vaccine/LLPC, Vaccine+Infection/LLPC, Vaccine/Non-LLPC, and Vaccine+Infection/Non-LLPC are 0.3192, 0.6529, 0.3446, and 0.9254, respectively (a); and 0.4933, 0.7301, 0.8004, and 0.7128, respectively (b). Subjects yielding sufficient ASC for LLPC and Non-LLPC subsets included.

We next compared the S2P BM ASC frequencies in SARS-CoV-2 infected subjects who were also vaccinated (n=5) with those who were only vaccinated with no self-reported infection (n=14). Between these two groups, we found no differences in S2P LLPC and Non-LLPC responses – stratified either by the time from the first (R^2^≤0.10, p≥0.32; Extended Data Figure 8a) or the last (R^2^≤0.19, p≥0.49; Extended Data Figure 8b) vaccine. Although the small number of samples made it difficult to draw definitive conclusions, these results suggest that SARS-CoV-2 infection may also fail to elicit BM LLPC.

### Declined serum S2P, not Flu, Tet, or total, IgG

**Fig. 3.**
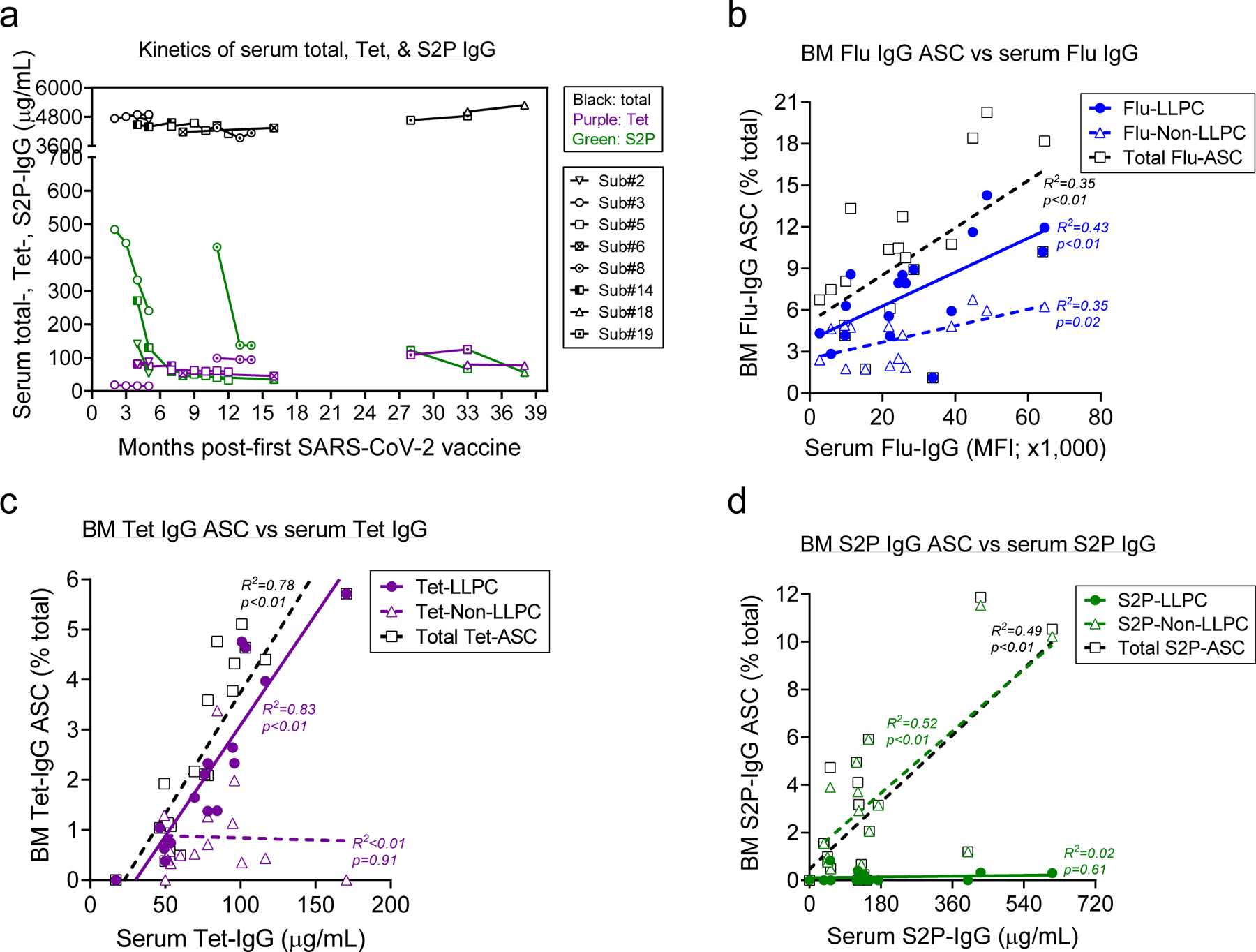
Vaccine-specific IgG levels in the serum: Kinetics and magnitude **(a) and correlation with BM IgG ASC responses (b**-**d).** Kinetics and magnitude of IgG titers from subjects (n=8) with at least two sequential serum samples (collected prior to the additional SARS-CoV-2 vaccines) (**a**). Serum IgG levels versus BM IgG LLPC, Non-LLPC, and total ASC responses for Flu (**b**), Tet (**c**), and S2P (**d**) specificities in all examined subjects (n=19). Sera collected within five months of the time of BM aspiration. For **b**-**d**, data were generated from eight different SARS-CoV-2 vaccinated subjects and correlations were assessed using simple linear regression analysis performed with GraphPad Prism (GraphPad Software). The exact *p* values for vaccine-specific LLPC, Non-LLPC, and total ASC are 0.0043, 0.0196, and 0.0075, respectively (**b**); <0.0001, 0.91, and <0.0001, respectively (**c**); and 0.6096, 0.0025, and 0.0008, respectively (**d**). Flu: influenza; Tet: tetanus. All serum samples tested at dilutions of 1:1,000–1:100,000 (total IgG) or 1:200–1:16,000 (antigen-specific IgG). For serum total and vaccine-specific IgG standard curves, see Supplementary Figure 1. For details of subjects and samples, see Table 1.

To assess the kinetics of serum antibodies, we measured total IgG as well as Flu, Tet, and S2P IgG responses up to 38 months after the first SARS-CoV-2 vaccine. From subjects with at least two sequential serum samples collected within five months of the time of BM aspiration (n=8) (Table 1), we observed a decline of S2P IgG titers in the serum within 3–6 months post-first SARS-CoV-2 vaccine (Fig. 3a). One subject had a booster at seven months after the first SARS-CoV-2 vaccine (Sub#8) which showed a rise and a rapid fall in antibody titers. While total IgG and Flu and Tet IgG titers in the serum were relatively stable during the period of 38 months after the first SARS-CoV-2 vaccine in this cohort, serum S2P IgG levels declined within 3–6 months of vaccination unless boosted by additional SARS-CoV-2 vaccines.

### Correlation of serum S2P IgG and BM IgG Non-LLPC

To investigate the relationship between systemic antibodies and BM ASC responses, we analyzed the IgG titers in the serum and the frequencies of BM IgG ASC (which include LLPC, Non-LLPC, and the sum of both as the total vaccine-specific ASC). We saw a modest correlation between serum Flu IgG and total BM Flu IgG ASC (R^2^=0.35, p<0.01; Fig. 3b). When separating LLPC and Non-LLPC from the total vaccine-specific ASC, there were also modest correlations of serum Flu IgG titers with BM Flu IgG Non-LLPC (R^2^=0.35, p=0.02) or with LLPC (R^2^=0.43, p<0.01). Interestingly, for Tet, we observed a very strong correlation between serum IgG titers and BM IgG LLPC (R^2^=0.83, p<0.01) or total ASC (R^2^=0.78, p<0.01) but not Non-LLPC (R^2^<0.01, p=0.91) (Fig. 3c). On the contrary, for S2P, we found a significant correlation between serum IgG levels and BM IgG Non-LLPC or total ASC (R^2^=0.52, p<0.01 or R^2^=0.49, p<0.01, respectively) but no correlation for LLPC (R^2^=0.02, p=0.61) (Fig. 3d). Together, these results show that serum Tet and Flu but not S2P IgG levels largely correlate with the vaccine-specific BM IgG LLPC responses; in contrast, serum IgG levels for S2P specificity are associated with the S2P BM IgG Non-LLPC frequencies.

### Kinetic responses for IgG ASC in longitudinal BM aspirates

**Fig. 4.**
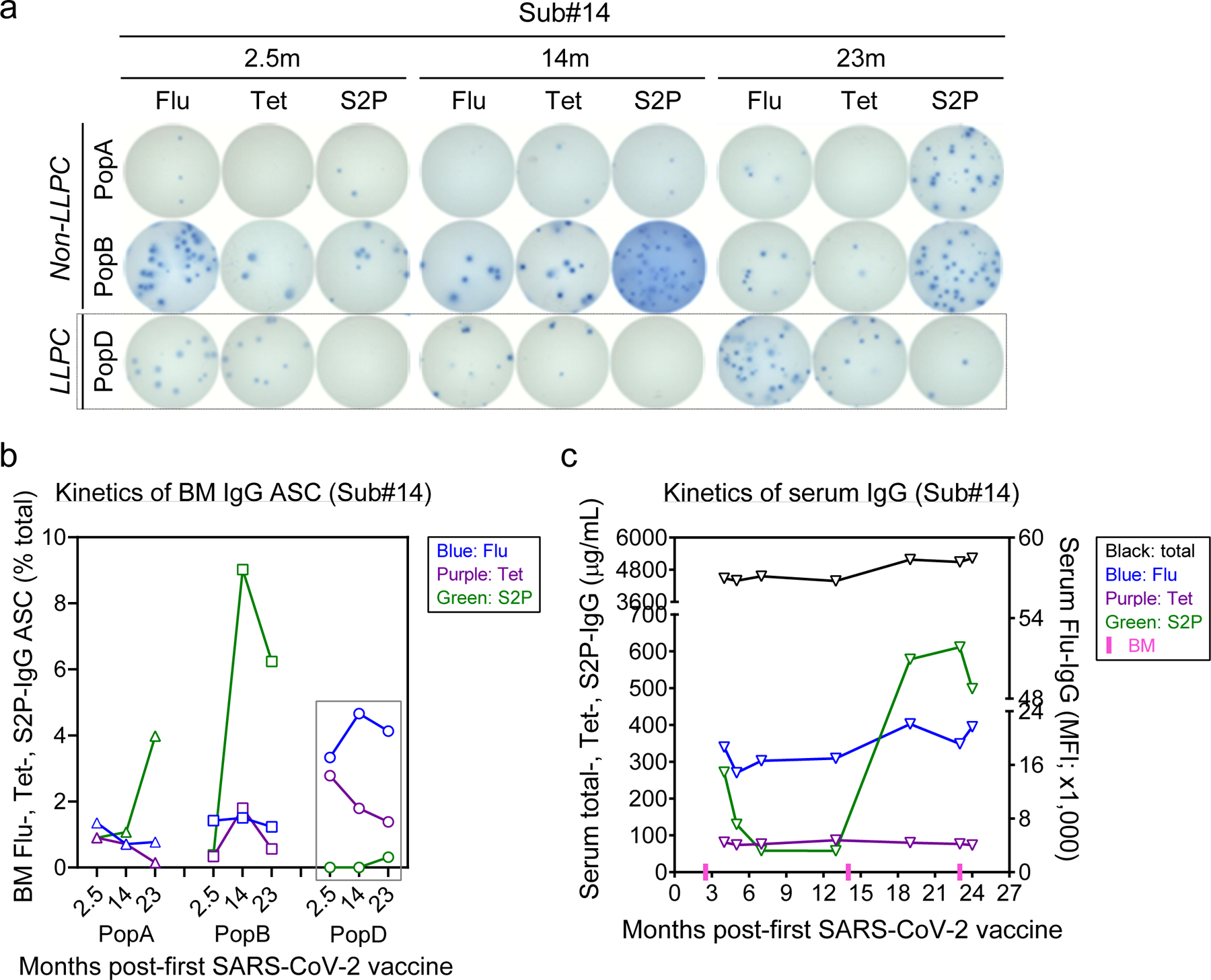
Kinetics and magnitude of BM IgG ASC responses and of total and vaccine-specific serum IgG levels in the subject who donated three longitudinal BM aspirates over two years (Sub#14). ELISpot scanned images (**a**). The numbers of input ASC that were incubated were ~21K, ~40K, and ~4.9K (2.5m); ~14K, ~12K, and ~3.8K (14m); and ~58K, ~22K, and ~7.2K (23m) for PopA, PopB, and PopD, respectively. Kinetics and magnitude of antigen-specific BM IgG ASC responses (**b**) and of total and antigen-specific IgG levels in the serum (**c**). Sera collected within 1–5 months of the time of BM aspiration. Part of Fig. 4c is reproduced from Fig. 3a for the purpose of kinetics comparison. Sub: subject; ~: counts provided by the sorters; K: 1,000; LLPC: long-lived plasma cell (dotted boxes in **a**,**b**); Flu: influenza; Tet: tetanus; m: month. All serum samples tested at dilutions of 1:1,000–1:100,000 (total IgG) or 1:200–1:16,000 (antigen-specific IgG). For serum total and vaccine-specific IgG standard curves, see Supplementary Figure 1. For details of subjects and samples, see Table 1.

We next assessed the IgG ASC kinetic responses in a subject (Sub#14) who provided three sequential BM aspirates over a period of 23 months. BM aspirates were taken 2.5, 14, and 23 months after the first SARS-CoV-2 vaccine (Table 1). Seven serum samples were collected within months of each BM aspiration. Each BM aspirate provided >3,000 FACS sorted ASC in each subset. Again, total IgG ASC were detected in all BM PopA, PopB, and PopD. We observed an increase in the frequencies of S2P IgG ASC in PopA and PopB at 14-month (1.07% and 9.02%, respectively) and 23-month (3.98% and 6.24%, respectively), compared to the first time-point (0.90% and 0.38%, respectively) (Fig. 4a,b). However, in PopD, there were no S2P IgG ASC detected at the first two time-points and only 0.31% at the last one (23-month). Notably, at the earliest time-point (2.5-month), the highest S2P IgG ASC frequency was observed in PopA, then at both later time-points (14-month and 23-month), it was highest in PopB. In all, regardless of time-points, the S2P ASC frequencies were always higher in PopA and PopB compared to PopD (even at 23-month). We observed the highest Flu and Tet frequencies in PopD, followed by PopB, and lowest in PopA. Interestingly, the Flu and Tet BM ASC frequencies were quite consistent over the course of two years.

In the serum, as expected, total IgG as well as Flu and Tet IgG levels were stable during the examined time periods (Fig. 4c). In contrast, after an initial decline within 3–6 months after SARS-CoV-2 vaccination, S2P IgG titers remained at low levels for about seven months. They then increased significantly, corresponding to the high frequencies of S2P PopB in the BM at 14-month and 23-month (Fig. 4b). Importantly, this increase occurred without any additional SARS-CoV-2 vaccination and stayed elevated for about six months, suggesting asymptomatic and/or unreported infections.

**Extended Data Fig. 9:**
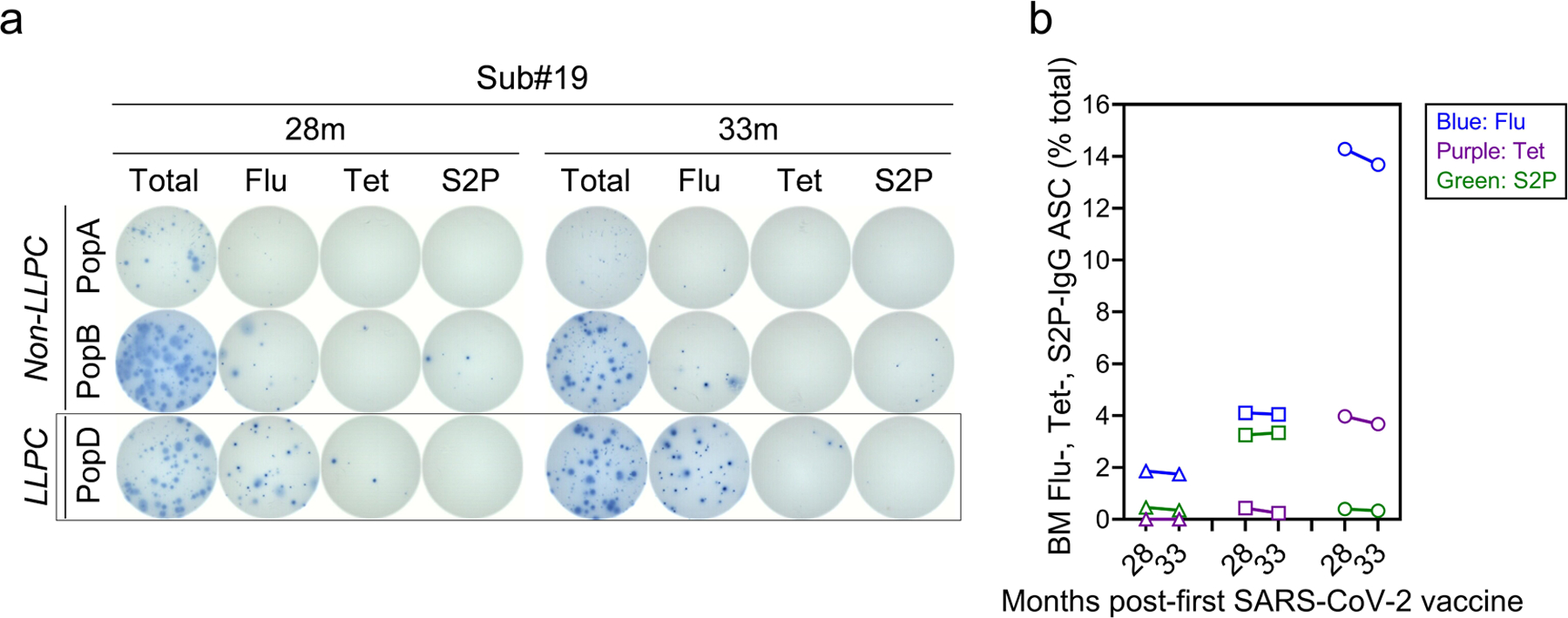
Kinetics of vaccine-specific IgG ASC responses in the subject who donated two sequential BM aspirates (at 28 and 33 months after the first SARS-CoV-2 vaccine).</P/>(a) ELISpot scanned images. The numbers of input ASC incubated were ~3.04K, ~1.27K, and ~0.88K (28m, total), ~15.20K, ~7.61K, and ~2.65K (28m, vaccine-specific); and ~1.56K, ~1.02K, and ~0.75K (33m, total), and ~18.67K, ~7.12K, and ~2.98K (33m, vaccine-specific), for PopA, PopB, and PopD, respectively. (b) Vaccine-specific IgG ASC response kinetics. ~: counts provided by the sorters; K: 1,000; LLPC: long-lived plasma cell (box); Flu: influenza; Tet: tetanus; m: month. For details of the subject, see Table 1.

In a second subject (Sub#19) with two sequential BM aspirates collected at 28-month and 33-month after the first SARS-CoV-2 vaccine (Table 1), the vaccine-specific IgG ASC responses in the BM were quite similar: the highest frequency of S2P ASC were found in PopB (3.25%−3.34%), followed by PopA (0.46%−0.35%), and PopD (0.40%−0.33%) (Extended Data Figure 9a,b). During this period, Flu and Tet BM ASC responses remained stable with the highest in PopD (Flu: 14.28% and 13.68%, and Tet: 3.97% and 3.67%, respectively) compared to PopB (Flu: 4.11% and 4.05%, and Tet: 0.43% and 0.24%, respectively) and PopA (Flu: 1.86% and 1.74%, and Tet: 0% and 0%, respectively). Thus, analysis of longitudinal BM aspirates demonstrates that S2P BM IgG ASC responses were consistently higher in PopA and PopB compared to PopD, suggesting S2P ASC are not established in the BM LLPC compartment after almost three years since the primary SARS-CoV-2 mRNA vaccination.

### Few subjects show S2P BM LLPC at low frequencies

**Fig. 5.**
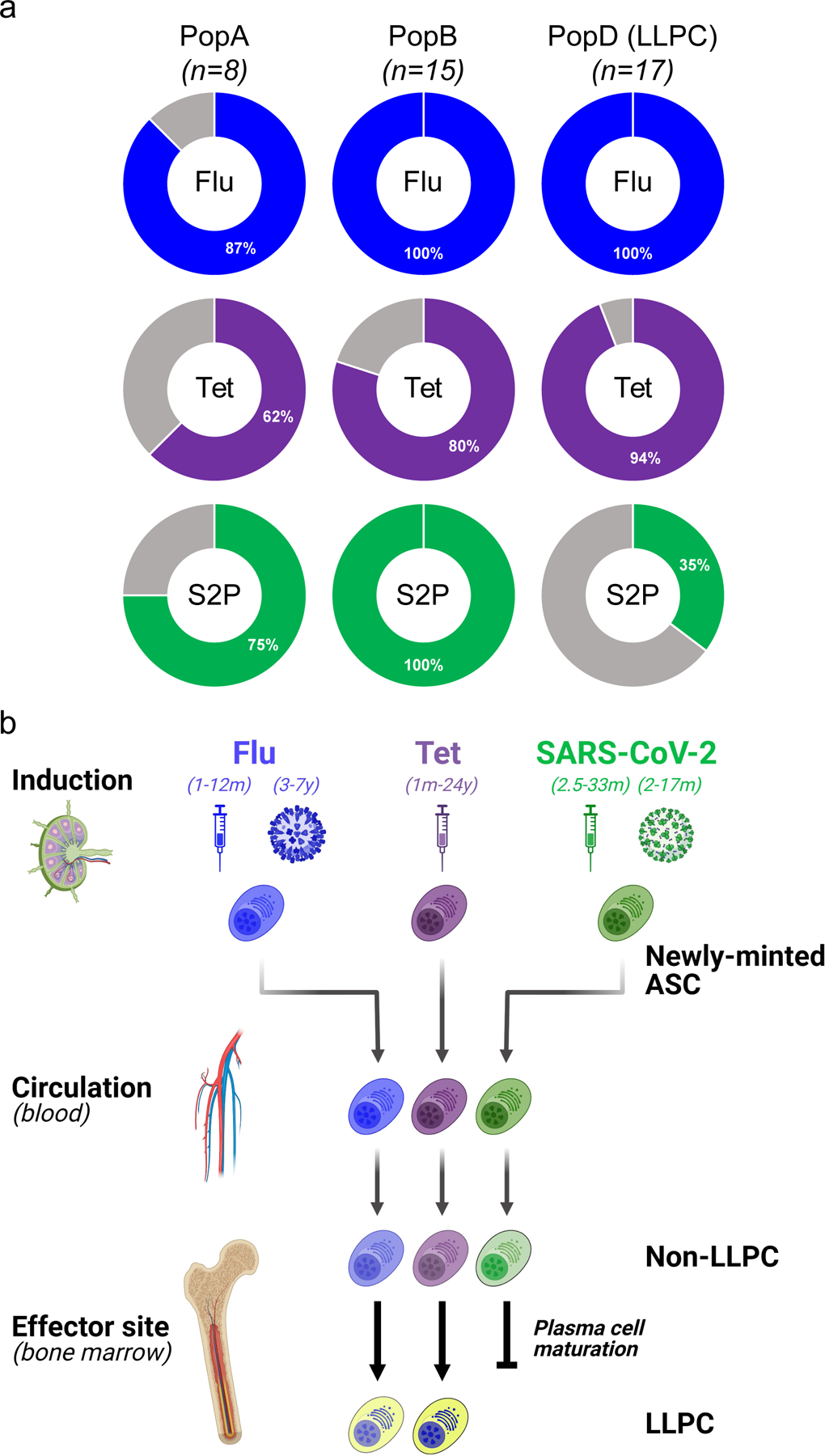
Antigen specificity strata of all individuals examined for each BM ASC subset (**a**). *n*, number of BM donors. **Graphical summary** (**b**). The majority of SARS-CoV-2 plasma cells are not established from the BM LLPC compartment 33 months after mRNA vaccination. Created with BioRender.com.

Finally, when we calculated the number of individuals with S2P-positive responses for each BM ASC subset, S2P IgG ASC were easily detected in PopA in 6/8 (75%) individuals and in PopB, in all 15/15 (100%) subjects (Fig. 5a). Only 6/17 (35.29%) subjects had S2P IgG ASC in PopD, and all were extremely low frequencies despite four or five doses of the vaccine and multiple known SARS-CoV-2 infections. As expected, nearly all subjects had easily detectable Flu and Tet specificities in PopD: 17/17 (100%) and 16/17 (94.12%) respectively. Altogether, durable serologic immune response correlates well with the abundance of Flu and Tet BM ASC in PopD while short-lived serologic antibody responses to SARS-CoV-2 mRNA vaccines may be explained by the exclusion of S2P ASC from this compartment (summarized in Fig. 5b).

## Discussion

In this study, we show that SARS-CoV-2 ASC in the BM are largely excluded from the LLPC compartment. This phenomenon is in stark contrast to Flu and Tet specificities which are inherent to the BM LLPC. These results highlight the importance of BM maturation programs, where an early-minted ASC undergoes dramatic morphological, transcriptional, and epigenetic modifications together with metabolic alterations, to undergo final maturation steps to become a LLPC^5,11^. Increased Ig transcripts^12^, increased unfolded protein response (UPR)^35^, anti-apoptotic^11^, and autophagy^36^ programs list a few of the pathways involved in ASC maturation^37^. Because this progression is arduous, not all the new arrivals can ultimately complete the entire LLPC process. Thus, dissecting the detailed mechanisms of the LLPC maturation programs will be important.

At one time, it was thought that all human ASC had the potential to become LLPC by simply migrating to environments rich in survival factors. However, recent evidence shows how imprinting of an early-minted ASC at the time of priming in addition to terminal maturation in survival niches endows particular properties for durability. LLPC are thought to come from memory B cells (mBC)^38^, especially mBC with FcRL5^+^ T-bet^+39^, but with SARS-CoV-2 mRNA vaccines, they fail to imprint these LLPC programs even 33 months after the vaccine. Thus, a longer tincture of time is unlikely to fill the LLPC subset, but more studies are needed.

There are two explanations for the abundant S2P specificity in PopA at 23 months as well as PopB at 14 and 23 months in the patient with sequential BM aspirates two years after the vaccine. Conventionally, PopA and PopB are the result of more recent immune responses and so breakthrough asymptomatic infections^40–42^ (which were well-described with the emergence of the highly transmissible Omicron variants^43–45^) temporally close to the corresponding BM sampling may explain these higher S2P frequencies. Nonetheless, even 23 months after vaccination and infection(s), S2P ASC still cannot fill the BM LLPC compartment. A second explanation is that lymph node S2P ASC, a product of ongoing germinal center (GC) reactions that can last for six months after vaccination^30,31^ continue to migrate to the BM. However, this argument still emphasizes the fact that even two years after the vaccine, PopB cannot differentiate into LLPC even with ongoing GC.

Our results are consistent with recent BM studies by Tehrani et al.^46^ demonstrating that most SARS-CoV-2 spike-specific ASC are detectable in the CD19^+^ compartments after SARS-CoV-2 infection alone. In this study, BM sampling occurred only 5–8 months post-illness and not up to three years as in our study and they used frozen BM ASC with limited viability upon thawing^46^. Also, the authors did not include longitudinal samples, IgA isotypes, Flu specificity, or PopA. Nonetheless, 5–8 months after infection alone, SARS-CoV-2 ASC still appear to be absent from the BM LLPC compartment, similar to our findings after vaccination.

**Extended Data Fig. 10:**
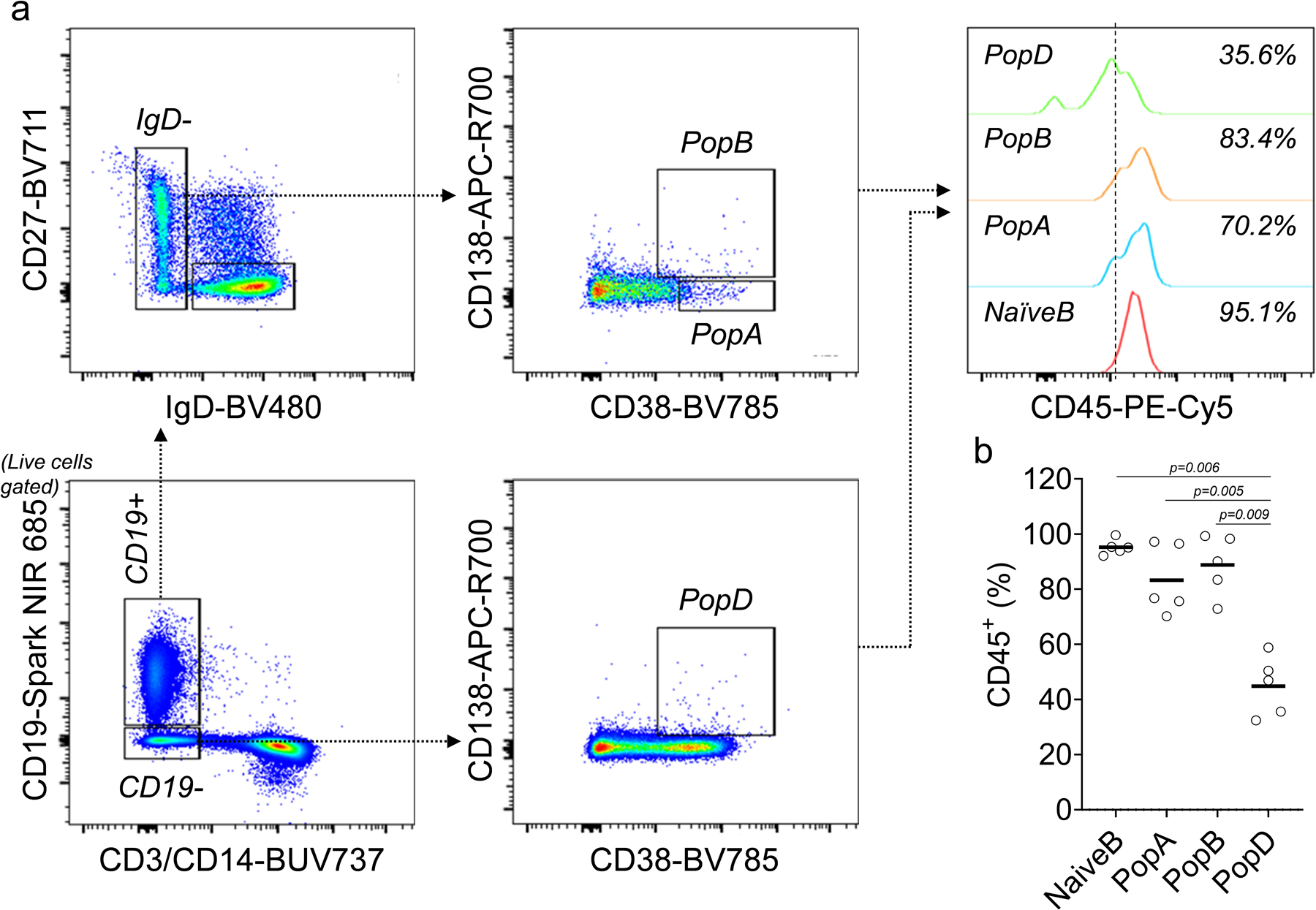
Downregulation of CD45 in LLPC (PopD).</P/>(a) Representative FACS gating strategy and CD45 staining for BM ASC subsets. For details on BM ASC gating, see Fig. 1b. For the antibody panels, see Methods. (b) CD45 staining is downregulated in PopD. Each circle represents an individual healthy BM donor. Data were generated from five different healthy BM donors. Statistic comparisons between any two CD45+ subsets were assessed using Student’s t-test (two-tailed unpaired t-test) in Excel (Microsoft) and differences were considered significant at p values less than 0.05. Shown are p values from comparisons with PopD; for p values from comparisons between other subsets, see Supplementary Table 5.

In another flow cytometry-based BM study, Schulz et al. found predominate SARS-CoV-2 S1-specific responses in the BM CD19^+^ ASC compartment after 17 months after vaccination^47^. The authors noted some specificity in the CD19^neg^ ASC compartment and concluded they are long-lived. However, these specific ASC are notably in the CD45^+^ (of CD19^neg^) ASC subset^47^, and the majority of LLPC demonstrate downregulation of CD45^10,48,49^ (Extended Data Figure 10). In concordance with our findings, in Schulz et al.^47^, the CD19^neg^CD45^neg^ subset, which includes the majority of our previously defined LLPC, also excludes SARS-CoV-2 responses. Hence, the *bona fide* LLPC, which may be a subset of the CD19^neg^ BM ASC population, likely harbors Flu, Tet, measles, and mumps specificities^10^ but excludes SARS-CoV-2 responses.

We cannot rule out the possibility where a subset of PopB may be an intermediary population on the road to LLPC maturation. Our previous single-cell transcriptional data showed that the most mature BM ASC clusters with aggregated LLPC also contain PopB^12^. Thus, simple surface markers CD19 and CD138 may be too blunt to dissect the heterogeneity of PopB, which includes new arrivals as well as early mature subsets. Ultimately, dissection into the transcriptional and epigenetic differences in Tet versus S2P PopB (CD19^+^CD138^+^) may reveal important mechanistic differences in the formation of long-lived ASC.

Although the emergence of new SARS-CoV-2 variants of confounded serum protection, we focused on responses against the original virus and the wildtype vaccines, knowing that they rapidly wane within 3–6 months regardless of the vaccine platform (mRNA or adenovirus (Ad) vectors)^3,4^. Interestingly, the Ad vectors persist for weeks, yet specific humoral immunity is also short-lasting^3,4^. Given both the mRNA and Ad vector vaccine platforms induce strong GC reactions and interactions with Tfh cells, the mechanisms underlying their failure to generate LLPC are even more puzzling^2^ and suggest dysfunction in the maturation process in the BM.

Could the limited durability of neutralizing antibody responses be due to the widely-spaced structural nature of the spike protein itself and thus limited only to coronavirus vaccines? Coronaviruses lack highly repetitive organized structures or pathogen-associated structural patterns^50^. Most RNA viruses that induce long-lasting antibody immunity have on their surface rigid repetitive structures spaced at 5–10nm^51^. For coronaviruses, the long spike proteins are embedded in a fluid membrane, which are often loosely floating and widely spaced at 25nm apart^50^. Therefore, the inherent nature of the spike protein itself may be an issue in B cell activation^51^ since neutralizing antibody responses to seasonal human coronaviruses, as well as to SARS-CoV-1 and MERS-CoV, are also short-lived^2^.

There are limitations in our study. First, our sample size is relatively small especially of those after both vaccination and infection. Second, the infections were self-reported symptoms that warranted testing, so any asymptomatic infections were not confirmed. Third, primary BM ASC are rare cell types and BM aspirates are difficult to obtain and interrogate; thus, not all samples provided sufficient cells for each BM subset. Fourth, we had limited longitudinal and sequential samples with the longest at 33 months since the first SARS-CoV-2 vaccine. Lastly, in this cohort, the modest correlation between serum Flu IgG and total BM Flu IgG ASC may not only reflect Flu-specific responses elicited by the last Flu vaccine but also exhibit cross-reactivity to older Flu strains^23^. Thus, it would also be important to assess the BM compartments decades after the primary vaccines as new variant SARS-CoV-2 viruses continue to emerge and circulate.

In conclusion, the holy grail of vaccinology is the generation of LLPC. Our findings demonstrate the exclusion of SARS-CoV-2 specificity in the BM LLPC and the need to improve durability of the mRNA vaccines. Whether optimizing vaccine regimens or immunization schedules, engineering different spike proteins, or formulating vaccine adjuvants and delivery systems will need better understanding.

## Methods

### Healthy human subjects.

A total of 22 BM aspirate samples were obtained from 19 healthy adult donors who self-reported to receive influenza, Tdap, and COVID-19 primary and booster vaccines. Serum samples were also collected from all the subjects (1–7 draws/subject) within five months (before and/or after) of BM aspiration. Select demographic and clinical characteristics of the subjects can be seen in Fig. 1a. Detailed information on BM subjects, BM aspirates, and serum samples can be found in Table 1. For antigen optimization and selection, peripheral blood samples were obtained from 64 healthy subjects who received vaccines for influenza, Tdap, or COVID-19 (the 3^rd^, 4^th^, or 5^th^ dose) at 5–7 days prior to sample collection. For CD45 flow cytometric staining, BM aspirates from an addition of five healthy BM subjects were obtained, stained, acquired, and analyzed.

Written informed consent was obtained from all subjects. Samples were collected over three years to be confident the results were statistically significant to distinguish different biological antigen-specific BM subsets. We recruited adult subjects who are healthy as defined by a health survey with no history of autoimmune, renal, liver, cardiopulmonary, and vascular disease. Patients with history of malignancy, transplant, HIV, hepatitis C, or those on immunosuppressive therapies are also excluded. The participants were remunerated for the time and inconvenience. All research was approved by the Emory University Institutional Review Board Committee (Emory IRB numbers IRB00066294 and IRB00057983) and was performed in accordance with all relevant guidelines and regulations.

### Purification of blood and BM ASC.

Isolation of peripheral blood mononuclear cells (PBMC) and BM mononuclear cells (BMMC) was performed according to our established procedure^16^. Briefly, mononuclear cells were isolated by Ficoll density gradient centrifugation and enriched by either a commercial human Pan-B cell enrichment kit (that removes cells expressing CD2, CD3, CD14, CD16, CD36, CD42b, CD56, CD66b, CD123, and glycophorin A) (StemCell Technologies) or a custom-designed negative selection cell isolation kit (that removes cells expressing CD3, CD14, CD66b, and glycophorin A) (StemCell Technologies) to limit sorting time and pressure on fragile ASC. For the antibody-secreting cell sorting panels, cell enriched fractions from blood or bone marrow aspirates were stained with the following anti-human antibodies: IgD–FITC (Cat. #555778; BD Biosciences) at 1:5 dilution or IgD-BV480 (Cat. #566138; BD Biosciences) at 1:5 dilution, CD3-BV711 (Cat. #317328; BioLegend) at 1:20 dilution or CD3-BUV737 (Cat. #612750; BD Biosciences) at 1:20 dilution, CD14-BV711 (Cat. #301838; BioLegend) at 1:20 dilution or CD14-BUV737 (Cat. #612763; BD Biosciences) at 1:20 dilution, CD19-PE-Cy7 (Cat. #560911; BD Biosciences) at 1:5 dilution or CD19-Spark NIR 685 (Cat. #302270; BioLegend) at 1:5 dilution, CD38-V450 (Cat. #561378; BD Bioscience) at 1:20 dilution or CD38-BV785 (Cat. #303530; BioLegend) at 1:20 dilution, CD138-APC (Cat. #130–117-395; Miltenyi Biotech) at 1:20 dilution or CD138-APC-R700 (Cat. #566050; BD Biosciences) at 1:20 dilution, CD27-APC-e780 (Cat. #5016160; eBiosciences) at 1:20 dilution or CD27-BV711 (Cat. #356430; BioLegend) at 1:20 dilution, and LiveDead (Cat. #L34966; Invitrogen) at 1:600 dilution or Zombie NIR Fixable Viability Kit (Cat. #423106; BioLegend) at 1:500 dilution.

Fresh blood ASC as well as BM ASC subsets, which included PopA, PopB, and PopD (LLPC), were purified using FACS-based sorting^10,16^. ASC subsets were sorted on a BD FACSAria II using a standardized sorting procedure with rainbow calibration particles to ensure consistency of sorts among individuals. ASC subsets were sorted as^10,11,16^: Blood ASC (IgD^−^CD27^hi^CD38^hi^), BM ASC (IgD^−^CD19^+^CD38^hi^), PopA (CD19^+^IgD^−^D38^hi^CD138^−^), PopB (CD19^+^IgD^−^CD38^hi^CD138^+^), and PopD (CD19^−^IgD^−^CD38^hi^CD138^+^). Sorted ASC populations were generally 93–99% pure (except for PopA whose purity was usually 60–75%).

### Antigen selection for Ig immunoassays.

The following antigens were used for vaccine-specific IgG and IgA capturing: quadrivalent influenza vaccine 2019–20, 2020–21, 2021–22, or 2023–24 (Fluarix Quadrivalent Influenza Vaccine 2019–20, 2020–21, 2021–22, or 2023–24 Formula, respectively; GSK Biologicals/ABO Pharmaceuticals; Afluria® Quadrivalent (Seqirus); or Fluzone® Quadrivalent (Sanofi Pasteur)), tetanus toxoid, *Clostridium tetani* (Calbiochem/Millipore Sigma or Fina Biosolutions), and SARS-CoV-2 S2P (recombinant SARS-CoV-2 soluble spike trimer protein, Lot #P210721.02; Protein Expression Laboratory, Frederick National Laboratory for Cancer Research (FNLCR), Frederick, MD). For relative quantitation of antigen-specific antibody titers, standard curves were generated using monoclonal antibody (mAb) standards of anti-tetanus toxin mAb (clone #TetE3; The Native Antigen Company) and SARS-CoV-2-reactive (spike RBD) mAb (Abeomics). For determining the concentrations of total IgG, purified human IgG (ChromePure human IgG, JacksonImmuno Research Laboratories) was used as a standard.

### Blood and BM ASC bulk cultures and ELISpot assays.

Human ASC cultures were conducted in MSC (mesenchymal stromal/stem cells) secretome (ASC survival medium) and in hypoxic conditions (2.5% O_2_) at 37°C^16^. This culture system is called plasma cell survival system (PCSS)^52^. IgG and IgA secretion of cultured ASC was assessed by ELISpot assays, which quantitated IgG- and IgA-secreting cells, respectively. These assays used goat anti-human IgG or IgA for total IgG or IgA capturing, respectively, and alkaline phosphatase-conjugated goat anti-human IgG or IgA, respectively, for detection, and were performed according to our established procedure^16^. ELISpot data was collected using the Cellular Technology Limited system (CTL), which run ImmunoSpot 5.0.9.21 software.

### Multiplex bead binding assays.

Multiplex bead binding assays (MBBA) were performed on the supernatants collected from culture of BM ASC purified from eight individuals who provided sufficient post-sort cells for all three subsets as well as from serum samples drawn from all 19 subjects (10 of which provided 2–7 sequential sera) (Table 1). For total IgG, biotinylated goat anti-human IgG (Southern Biotech) was conjugated to avidin-coupled MagPlex-avidin microspheres of spectrally distinct regions^53^. For vaccine-specific MBBA, antigens were conjugated to MagPlex microspheres (Luminex) of spectrally distinct regions via standard carbodiimide coupling procedure^34^. MBBA were performed using a FLEXMAP 3D instrument (Luminex)^34^. All viral protein coupled microspheres were tested together as a combined multiplex antigen-specific immunoassay and all anti-human Ig coupled microspheres were tested together as a combined multiplex total Ig immunoassay. Median fluorescent intensity (MFI) using combined or individual detection antibodies was measured using the xPONENT 4.3 software (Luminex) at enhanced PMT setting. The net MFI was obtained by subtracting the background value. The culture supernatant MFI values were normalized to the relative IgG concentrations (pg/mL) based on the total human IgG standard curves, followed by normalization of these resultant IgG concentrations (pg/mL) to the ASC input numbers and duration of culture (day). The MFI normalization and binding curves were performed based on the equations shown in Extended Data Figure 4 and Supplementary Figure 1. Data were expressed as the percents or ratios of the titers of antigen-specific IgG to those of total IgG (BM ASC culture supernatants) or as IgG concentrations (µg/mL) (serum total IgG and Tet and S2P IgG). Since the Flu specificities used (vaccines) had four antigens in combination and there were no mAb standards, we used MFI values as a semi-quantitative measure for assessment of Flu IgG levels in the serum. All the BM ASC culture supernatants were collected after one day in culture of off-sorter BM ASC subsets and were tested undiluted (neat) or 1:2 diluted – except for the total IgG titrations which were also assayed at further dilutions. All sera were assayed at dilutions of 1:1,000–1:100,000 (total IgG) or 1:200–1:16,000 (antigen-specific IgG).

### CD45 BM ASC flow cytometry.

BMMC were isolated according to our established procedure^16^ and stained with the following anti-human antibodies: IgD-BV480 (Cat. #566138; BD Biosciences) at 1:160 dilution, CD3-BUV737 (Cat. #612750; BD Biosciences) at 1:160 dilution, CD14-BUV737 (Cat. #612763; BD Biosciences) at 1:160 dilution, CD19-Spark NIR 685 (Cat. #302270; BioLegend) at 1:160 dilution, CD38-BV785 (Cat. #303530; BioLegend) at 1:160 dilution, CD138-APC-R700 (Cat. #566050; BD Biosciences) at 1:320 dilution, CD27-BV711 (Cat. #356430; BioLegend) at 1:40 dilution, and CD45-PE-Cy5 (Cat. #304009; BioLegend) at 1:160 dilution. Samples were run on a Cytek’s Aurora Spectral Flow Cytometer using Cytek SpectroFlo software (v3.0; Cytek Biosciences) and analyzed with the FlowJo v10.8.1 software (FlowJo, LLC).

### Statistics.

Statistics were assessed using Student’s t-test (two-tailed unpaired t-test) in Excel (Microsoft) and differences were considered significant at *p* values less than 0.05. Correlations were assessed using simple linear regression analysis performed with GraphPad Prism (v8.4.2; GraphPad Software). No adjustments were made for multiple comparisons.

## Extended Data

## Supplementary Material

Lee_Supplementary_Figure_1

Lee_Supplementary_Table_1

Lee_Supplementary_Table_2

Lee_Supplementary_Table_3

Lee_Supplementary_Table_4

Lee_Supplementary_Table_5

## Figures and Tables

**Table 1.** BM subjects and BM samples.

Year &		2020				2021												2022												2023												2024			mRNA vaccine		Demographic profile		
Subject ID		Pre-Sept	Oct	Nov	Dec	Jan	Feb	Mar	Apr	May	Jun	Jul	Aug	Sept	Oct	Nov	Dec	Jan	Feb	Mar	Apr	May	Jun	Jul	Aug	Sept	Oct	Nov	Dec	Jan	Feb	Mar	Apr	May	Jun	Jul	Aug	Sept	Oct	Nov	Dec	Jan	Feb	Mar	Pfizer	Moderna	Age	Sex	Race
###	Sub#1	Tet (08/2018)	Flu	Inf*				V1	V2; Inf*		BM														*V3*							*V4*													√		31	F	Caucasian
	Sub#2	Tet (05/2017)	Flu					V1	V2		BM					*V3*		*V4*																											√		26	M	Caucasian
	Sub#3	Tet (11/2011)	Flu					V1	V2		BM				*Flu*														*V3*																	√	22	F	Caucasian
	Sub#4	Tet (pre-2013)	Flu					V1	V2				BM			*V3*	*Inf* *						*V4*				*V5; Flu*				*Tet*														√	√	56	F	Asian
	Sub#5	Tet (pre-2008)	Flu				V1	V2							Tet; Flu	BM		*V3*																												√	23	F	Asian
	Sub#6	Tet (pre-2011)	Flu		V1	V2									Flu	BM								*V3*			*V4*		*V5*																√		56	F	Asian
	Sub#7	Tet (06/2013)				Inf*			V1	V2				Flu		V3; BM		*Inf* *																											√		26	F	Caucasian
###	Sub#8	Tet (pre-2011)		Flu					V1	V2				Tet	V3	Flu						BM																								√	42	M	Caucasian
	Sub#9	Tet (pre-2010)	Tet			V1	V2								Flu						V4	BM					*Flu*																			√	55	F	Caucasian
	Sub#10	Tet (pre-2011)		Flu				V1; V2					Tet		Flu		V3						BM				*V4*																			√	36	M	Caucasian
	Sub#11	Tet (pre-2012)	Flu				V1	V2						V3	Flu						V4			BM																					√		20	F	Black
###	Sub#12	Tet (pre-2010)		Tet; Flu		V1	V2								V3	Flu		Inf*						V4			Flu; V5			BM																√	63	F	Black
	Sub#13	Tet (pre-2010)	Tet	Flu					V1						Flu		V2										Flu		V4		BM														√		48	M	Caucasian
	Sub#14	Tet (pre-2010)							V1	V2	BM												BM								BM															√	63	M	Black
		Flu (pre-2020)																																															
	Sub#15	Tet (05/1999)		Flu				V1					V2			Flu											V3		Flu					BM											√		36	M	Caucasian
	Sub#16	Tet (09/2017)	Flu					V1	V2						Flu			Inf*						Inf*				V3; Flu						BM											√		65	F	Black
	Sub#17	Tet (pre-2012)		Flu				V1		V2						Flu		V3					Inf*						Tet; Flu									BM							√		30	F	Caucasian
	Sub#18	Tet (07/2009)		Flu			V1; V2									Flu											Flu	V3										Flu	BM						√		28	F	Asian
	Sub#19	Tet (06/2015)		Flu								V1			Flu												Flu	V2										Flu	BM					BM	√	√	30	M	Caucasian

* Inf, infected with SARS-CoV-2. Sub, subject; Tet, Flu: tetanus, influenza vaccination, respectively; V1-V5: dose 1–5 of SARS-CoV-2 vaccine; BM: bone marrow; F: female; M: male; Filled cell, serum drawn. *Italic*, occurred after BM aspirate. For multiple-aspirate subjects, age at the most recent BM aspirate.

## Data Availability

There are no restrictions on the availability of experimental data from and of unique materials used in this study. All the data generated and/or analyzed in this study are available from the corresponding author. All unique materials used are readily available from the corresponding author and Emory University.

## References

[1] WHO. https://covid19.who.int. Accessed 12/06/2023 (2023).

[2] Nguyen DC COVID-19 and plasma cells: Is there long-lived protection? Immunol Rev 309, 40–63, doi:10.1111/imr.13115 (2022). 35801537 PMC9350162

[3] Bhattacharya D Instructing durable humoral immunity for COVID-19 and other vaccinable diseases. Immunity 55, 945–964, doi:10.1016/j.immuni.2022.05.004 (2022). 35637104 PMC9085459

[4] Lasrado N & Barouch DH SARS-CoV-2 Hybrid Immunity: The Best of Both Worlds. J Infect Dis, doi:10.1093/infdis/jiad353 (2023). 37592872

[5] Nguyen DC Plasma cell survival: The intrinsic drivers, migratory signals, and extrinsic regulators. Immunol Rev 303, 138–153, doi:10.1111/imr.13013 (2021). 34337772 PMC8387437

[6] Nguyen DC Majority of human circulating IgG plasmablasts stop blasting in a cell-free pro-survival culture. Sci Rep 14, 3616, doi:10.1038/s41598-024-53977-2 (2024). 38350990 PMC10864258

[7] Turner JS SARS-CoV-2 infection induces long-lived bone marrow plasma cells in humans. Nature, doi:10.1038/s41586-021-03647-4 (2021). 34030176

[8] Kim W Germinal centre-driven maturation of B cell response to mRNA vaccination. Nature 604, 141–145, doi:10.1038/s41586-022-04527-1 (2022). 35168246 PMC9204750

[9] Prabhakaran M Adjuvanted SARS-CoV-2 spike protein vaccination elicits long-lived plasma cells in nonhuman primates. Sci Transl Med 16, eadd5960, doi:10.1126/scitranslmed.add5960 (2024). 38170789

[10] Halliley JL Long-Lived Plasma Cells Are Contained within the CD19(−)CD38(hi)CD138(+) Subset in Human Bone Marrow. Immunity 43, 132–145, doi:10.1016/j.immuni.2015.06.016 (2015). 26187412 PMC4680845

[11] Joyner CJ Generation of human long-lived plasma cells by developmentally regulated epigenetic imprinting. Life Sci Alliance 5, doi:10.26508/lsa.202101285 (2022). PMC8739272 34952892

[12] Duan M Understanding heterogeneity of human bone marrow plasma cell maturation and survival pathways by single-cell analyses. Cell Rep 42, 112682, doi:10.1016/j.celrep.2023.112682 (2023). 37355988 PMC10391632

[13] Liu X, Yao J, Zhao Y, Wang J & Qi H Heterogeneous plasma cells and long-lived subsets in response to immunization, autoantigen and microbiota. Nat Immunol 23, 1564–1576, doi:10.1038/s41590-022-01345-5 (2022). 36316480

[14] Robinson MJ Intrinsically determined turnover underlies broad heterogeneity in plasma-cell lifespan. Immunity 56, 1596–1612 e1594, doi:10.1016/j.immuni.2023.04.015 (2023). 37164016

[15] Mei HE A unique population of IgG-expressing plasma cells lacking CD19 is enriched in human bone marrow. Blood 125, 1739–1748, doi:10.1182/blood-2014-02-555169 (2015). 25573986

[16] Nguyen DC Factors of the bone marrow microniche that support human plasma cell survival and immunoglobulin secretion. Nat Commun 9, 3698, doi:10.1038/s41467-018-05853-7 (2018). 30209264 PMC6135805

[17] Nguyen DC, Joyner CJ, Sanz I & Lee FE Factors Affecting Early Antibody Secreting Cell Maturation Into Long-Lived Plasma Cells. Front Immunol 10, 2138, doi:10.3389/fimmu.2019.02138 (2019). 31572364 PMC6749102

[18] Tellier J & Nutt SL The secret to longevity, plasma cell style. Nat Immunol 23, 1507–1508, doi:10.1038/s41590-022-01340-w (2022). 36316478

[19] Amanna IJ, Carlson NE & Slifka MK Duration of humoral immunity to common viral and vaccine antigens. N Engl J Med 357, 1903–1915, doi:10.1056/NEJMoa066092 (2007). 17989383

[20] Krammer F The human antibody response to influenza A virus infection and vaccination. Nat Rev Immunol 19, 383–397, doi:10.1038/s41577-019-0143-6 (2019). 30837674

[21] Halasa NB, Gerber MA, Chen Q, Wright PF & Edwards KM Safety and immunogenicity of trivalent inactivated influenza vaccine in infants. J Infect Dis 197, 1448–1454, doi:10.1086/587643 (2008). 18444800 PMC3773726

[22] Bodewes R Prevalence of antibodies against seasonal influenza A and B viruses in children in Netherlands. Clin Vaccine Immunol 18, 469–476, doi:10.1128/CVI.00396-10 (2011). 21209157 PMC3067385

[23] Kucharski AJ Estimating the life course of influenza A(H3N2) antibody responses from cross-sectional data. PLoS Biol 13, e1002082, doi:10.1371/journal.pbio.1002082 (2015). 25734701 PMC4348415

[24] Hancock K Cross-reactive antibody responses to the 2009 pandemic H1N1 influenza virus. N Engl J Med 361, 1945–1952, doi:10.1056/NEJMoa0906453 (2009). 19745214

[25] (CDC), C. f. D. C. a. P. Serum cross-reactive antibody response to a novel influenza A (H1N1) virus after vaccination with seasonal influenza vaccine. MMWR Morb Mortal Wkly Rep 58, 521–524 (2009). 19478718

[26] Skountzou I Immunity to pre-1950 H1N1 influenza viruses confers cross-protection against the pandemic swine-origin 2009 A (H1N1) influenza virus. J Immunol 185, 1642–1649, doi:10.4049/jimmunol.1000091 (2010). 20585035 PMC4457446

[27] Fisman DN Older age and a reduced likelihood of 2009 H1N1 virus infection. N Engl J Med 361, 2000–2001, doi:10.1056/NEJMc0907256 (2009). 19907052

[28] Nachbagauer R Defining the antibody cross-reactome directed against the influenza virus surface glycoproteins. Nat Immunol 18, 464–473, doi:10.1038/ni.3684 (2017). 28192418 PMC5360498

[29] Yu X Neutralizing antibodies derived from the B cells of 1918 influenza pandemic survivors. Nature 455, 532–536, doi:10.1038/nature07231 (2008). 18716625 PMC2848880

[30] Wrammert J Rapid cloning of high-affinity human monoclonal antibodies against influenza virus. Nature 453, 667–671, doi:10.1038/nature06890 (2008). 18449194 PMC2515609

[31] Lee FE Circulating human antibody-secreting cells during vaccinations and respiratory viral infections are characterized by high specificity and lack of bystander effect. J Immunol 186, 5514–5521, doi:10.4049/jimmunol.1002932 (2011). 21441455 PMC3726212

[32] Wrapp D Cryo-EM structure of the 2019-nCoV spike in the prefusion conformation. Science 367, 1260–1263, doi:10.1126/science.abb2507 (2020). 32075877 PMC7164637

[33] Odendahl M Generation of migratory antigen-specific plasma blasts and mobilization of resident plasma cells in a secondary immune response. Blood 105, 1614–1621, doi:10.1182/blood-2004-07-2507 (2005). 15507523

[34] Haddad NS One-Stop Serum Assay Identifies COVID-19 Disease Severity and Vaccination Responses. Immunohorizons 5, 322–335, doi:10.4049/immunohorizons.2100011 (2021). 34001652 PMC9190970

[35] Tellier J Blimp-1 controls plasma cell function through the regulation of immunoglobulin secretion and the unfolded protein response. Nat Immunol 17, 323–330, doi:10.1038/ni.3348 (2016). 26779600 PMC4757736

[36] Pengo N Plasma cells require autophagy for sustainable immunoglobulin production. Nature immunology 14, 298–305, doi:10.1038/ni.2524 (2013). 23354484

[37] Lam WY Metabolic and Transcriptional Modules Independently Diversify Plasma Cell Lifespan and Function. Cell Rep 24, 2479–2492 e2476, doi:10.1016/j.celrep.2018.07.084 (2018). 30157439 PMC6172041

[38] Palm AE & Henry C Remembrance of Things Past: Long-Term B Cell Memory After Infection and Vaccination. Front Immunol 10, 1787, doi:10.3389/fimmu.2019.01787 (2019). 31417562 PMC6685390

[39] Nellore A A transcriptionally distinct subset of influenza-specific effector memory B cells predicts long-lived antibody responses to vaccination in humans. Immunity 56, 847–863 e848, doi:10.1016/j.immuni.2023.03.001 (2023). 36958335 PMC10113805

[40] White EM Asymptomatic and Presymptomatic Severe Acute Respiratory Syndrome Coronavirus 2 Infection Rates in a Multistate Sample of Skilled Nursing Facilities. JAMA Intern Med 180, 1709–1711, doi:10.1001/jamainternmed.2020.5664 (2020). 33074318 PMC7573793

[41] Shang W Percentage of Asymptomatic Infections among SARS-CoV-2 Omicron Variant-Positive Individuals: A Systematic Review and Meta-Analysis. Vaccines (Basel) 10, doi:10.3390/vaccines10071049 (2022). PMC9321237 35891214

[42] El-Ghitany EM Asymptomatic versus symptomatic SARS-CoV-2 infection: a cross-sectional seroprevalence study. Trop Med Health 50, 98, doi:10.1186/s41182-022-00490-9 (2022). 36575501 PMC9792933

[43] Garrett N High Asymptomatic Carriage With the Omicron Variant in South Africa. Clin Infect Dis 75, e289–e292, doi:10.1093/cid/ciac237 (2022). 35353885 PMC9383623

[44] Christensen PA Signals of Significantly Increased Vaccine Breakthrough, Decreased Hospitalization Rates, and Less Severe Disease in Patients with Coronavirus Disease 2019 Caused by the Omicron Variant of Severe Acute Respiratory Syndrome Coronavirus 2 in Houston, Texas. Am J Pathol 192, 642–652, doi:10.1016/j.ajpath.2022.01.007 (2022). 35123975 PMC8812084

[45] Oran DP & Topol EJ Prevalence of Asymptomatic SARS-CoV-2 Infection : A Narrative Review. Ann Intern Med 173, 362–367, doi:10.7326/M20-3012 (2020). 32491919 PMC7281624

[46] Tehrani ZR Deficient Generation of Spike-Specific Long-Lived Plasma Cells in the Bone Marrow After Severe Acute Respiratory Syndrome Coronavirus 2 Infection. J Infect Dis, doi:10.1093/infdis/jiad603 (2024). PMC11272043 39052732

[47] Schulz AR SARS-CoV-2 specific plasma cells acquire long-lived phenotypes in human bone marrow. EBioMedicine 95, 104735, doi:10.1016/j.ebiom.2023.104735 (2023). 37556944 PMC10432952

[48] Mujtahedi SS Bone marrow derived long-lived plasma cell phenotypes are heterogeneous and can change in culture. Transpl Immunol 75, 101726, doi:10.1016/j.trim.2022.101726 (2022). 36183942

[49] Pellat-Deceunynck C & Bataille R Normal and malignant human plasma cells: proliferation, differentiation, and expansions in relation to CD45 expression. Blood Cells Mol Dis 32, 293–301, doi:10.1016/j.bcmd.2003.12.001 (2004). 15003821

[50] Bachmann MF, Mohsen MO, Zha L, Vogel M & Speiser DE SARS-CoV-2 structural features may explain limited neutralizing-antibody responses. NPJ Vaccines 6, 2, doi:10.1038/s41541-020-00264-6 (2021). 33398006 PMC7782831

[51] Slifka MK & Amanna IJ Role of Multivalency and Antigenic Threshold in Generating Protective Antibody Responses. Front Immunol 10, 956, doi:10.3389/fimmu.2019.00956 (2019). 31118935 PMC6504826

[52] Woodruff MC Response under pressure: deploying emerging technologies to understand B-cell-mediated immunity in COVID-19. Nat Methods 19, 387–391, doi:10.1038/s41592-022-01450-1 (2022). 35396475 PMC9703369

[53] Haddad NS Circulating antibody-secreting cells are a biomarker for early diagnosis in patients with Lyme disease. PLoS One 18, e0293203, doi:10.1371/journal.pone.0293203 (2023). 37922270 PMC10624293

