# Supplementary figures and images for: SARS-CoV-2 Plasma Cells are Not Durably Established in the Bone Marrow Long-Lived Compartment after mRNA Vaccination
Source: Nat Med. Author manuscript; Available in PMC 2025 Jan 23. (PMC11750719; doi:10.1038/s41591-024-03278-y)

Total-IgG

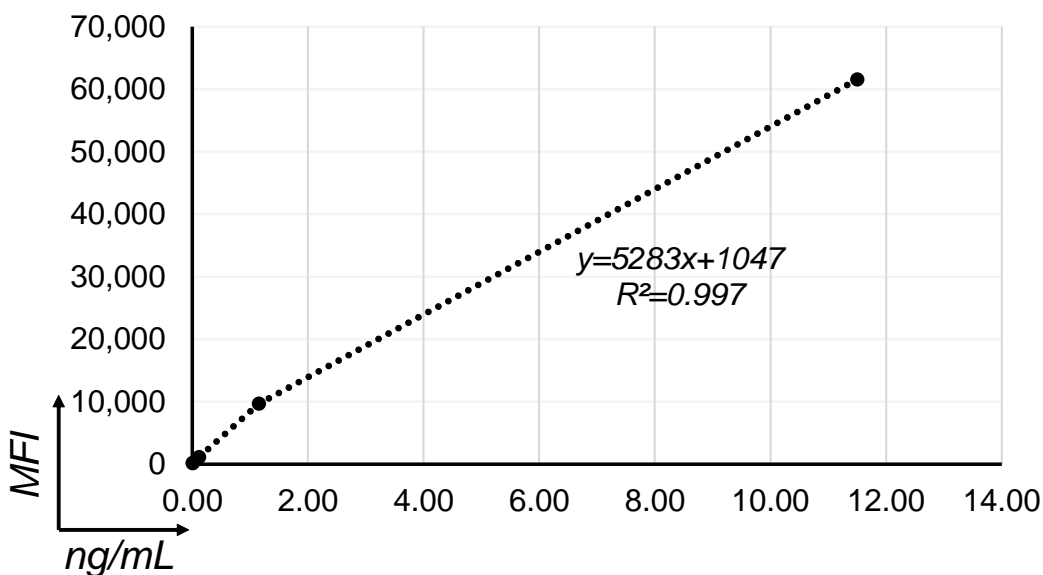

Tet-IgG

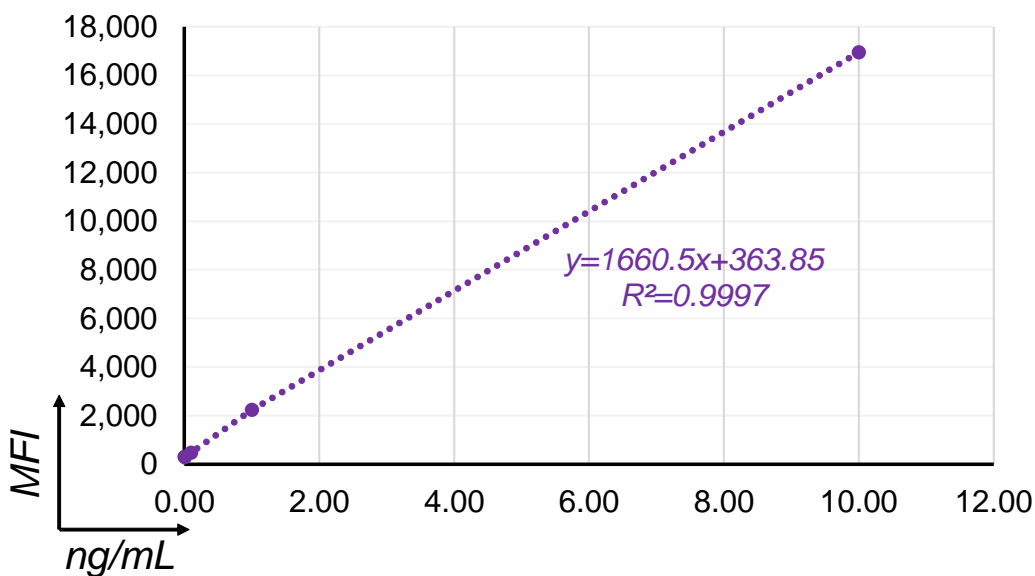

S2P-IgG

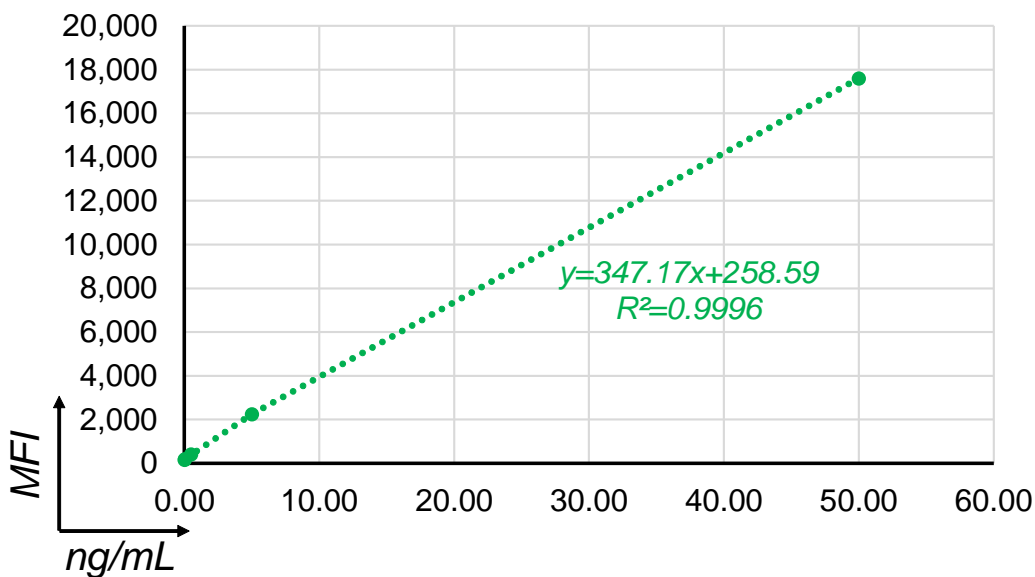

Supplement: Lee_Supplementary_Figure_1 [file NIHMS2035548-supplement-Lee_Supplementary_Figure_1.pdf]
